# Aggressive PitNETs and Potential Target Therapies: A Systematic Review of Molecular and Genetic Pathways

**DOI:** 10.3390/ijms242115719

**Published:** 2023-10-29

**Authors:** Simona Serioli, Ludovico Agostini, Alberto Pietrantoni, Federico Valeri, Flavia Costanza, Sabrina Chiloiro, Barbara Buffoli, Amedeo Piazza, Pietro Luigi Poliani, Maria Peris-Celda, Federica Iavarone, Simona Gaudino, Marco Gessi, Giovanni Schinzari, Pier Paolo Mattogno, Antonella Giampietro, Laura De Marinis, Alfredo Pontecorvi, Marco Maria Fontanella, Liverana Lauretti, Guido Rindi, Alessandro Olivi, Antonio Bianchi, Francesco Doglietto

**Affiliations:** 1 Division of Neurosurgery, Department of Medical and Surgical Specialties, Radiological Sciences and Public Health, University of Brescia, 25123 Brescia, Italy; marco.fontanella@unibs.it; 2 Facoltà di Medicina e Chirurgia, Università Cattolica del Sacro Cuore, 20123 Rome, Italy; ludovico.agostini01@icatt.it (L.A.); federicovaleri97@gmail.com (F.V.); flavia.costanza.fc@gmail.com (F.C.); simona.gaudino@policlinicogemelli.it (S.G.); marco.gessi@policlinicogemelli.it (M.G.); giovanni.schinzari@policlinicogemelli.it (G.S.); laura.demarinis@policlinicogemelli.it (L.D.M.); alfredo.pontecorvi@policlinicogemelli.it (A.P.); liverana.lauretti@policlinicogemelli.it (L.L.); guido.rindi@policlinicogemelli.it (G.R.); alessandro.olivi@policlinicogemelli.it (A.O.); antonio.bianchi@policlinicogemelli.it (A.B.); francesco.doglietto@unicatt.it (F.D.); 3 Department of Neurosurgery, Fondazione Policlinico Universitario Agostino Gemelli IRCCS, 00168 Rome, Italy; pierpaolo.mattogno@policlinicogemelli.it; 4 Department of Pathology, University of Brescia, 25123 Brescia, Italy; alpietra94@gmail.com; 5 Pituitary Unit, Division of Endocrinology and Metabolism, Fondazione Policlinico Universitario A. Gemelli, IRCCS, 00168 Rome, Italy; antonella.giampietro@policlinicogemelli.it; 6 Section of Anatomy and Physiopathology, Department of Clinical and Experimental Sciences, University of Brescia, 25121 Brescia, Italy; barbara.buffoli@unibs.it; 7 Department of Neuroscience, Neurosurgery Division, “Sapienza” University of Rome, 00185 Rome, Italy; amedeo.piazza@uniroma1.it; 8 Pathology Unit, Vita-Salute San Raffaele University, IRCCS San Raffaele, 20132 Milan, Italy; poliani.luigi@hsr.it; 9 Department of Neurologic Surgery, Mayo Clinic, Rochester, MN 55905, USA; periscelda.maria@mayo.edu; 10 Department of Otolaryngology/Head and Neck Surgery, Mayo Clinic, Rochester, MN 55905, USA; 11 Dipartimento di Scienze Biotecnologiche di Base, Cliniche Intensivologiche e Perioperatorie, Università Cattolica del Sacro Cuore, 20123 Rome, Italy; federica.iavarone@unicatt.it; 12 Fondazione Policlinico Universitario IRCCS “A. Gemelli”, 00168 Rome, Italy; 13 Department of Radiological Sciences, Institute of Radiology, Fondazione Policlinico Universitario A. Gemelli, IRCCS, 00168 Rome, Italy; 14 Neuropathology Unit, Fondazione Policlinico Universitario A. Gemelli, IRCCS, 00168 Rome, Italy; 15 Department of Oncology, Fondazione Policlinico Universitario A. Gemelli, IRCCS, 00168 Rome, Italy

**Keywords:** aggressive PitNETs, gene expression, invasive PitNETs, molecular pathway, precision medicine, target therapy

## Abstract

Recently, advances in molecular biology and bioinformatics have allowed a more thorough understanding of tumorigenesis in aggressive PitNETs (pituitary neuroendocrine tumors) through the identification of specific essential genes, crucial molecular pathways, regulators, and effects of the tumoral microenvironment. Target therapies have been developed to cure oncology patients refractory to traditional treatments, introducing the concept of precision medicine. Preliminary data on PitNETs are derived from preclinical studies conducted on cell cultures, animal models, and a few case reports or small case series. This study comprehensively reviews the principal pathways involved in aggressive PitNETs, describing the potential target therapies. A search was conducted on Pubmed, Scopus, and Web of Science for English papers published between 1 January 2004, and 15 June 2023. 254 were selected, and the topics related to aggressive PitNETs were recorded and discussed in detail: epigenetic aspects, membrane proteins and receptors, metalloprotease, molecular pathways, PPRK, and the immune microenvironment. A comprehensive comprehension of the molecular mechanisms linked to PitNETs’ aggressiveness and invasiveness is crucial. Despite promising preliminary findings, additional research and clinical trials are necessary to confirm the indications and effectiveness of target therapies for PitNETs.

## 1. Introduction

Pituitary adenomas, namely pituitary neuroendocrine tumors (PitNETs), are a heterogeneous group of neoplasms deriving from the neuroendocrine cell of the adeno-pituitary and represent 15% of all intracranial tumors [1,2]. Although most PitNETs are benign and respond to conventional treatment with an excellent outcome, up to 40% of cases display aggressive behavior, with an unpredictable and rapid evolution affecting the patient’s overall quality of life [3,4]. For this subgroup, the term “aggressive pituitary adenoma” was used; in 2017, the acronym PitNETs was suggested for all pituitary adenomas, highlighting the need to determine the benign or aggressive nature of each one of them and underscoring the heterogeneity of the biological and clinical behavior of these lesions [5]. According to the European Society of Endocrinology (ESE), aggressive PitNETs are characterized by propagation to extrasellar regions (traditionally defined in the parasellar area according to Knosp’s modified classification), rapid tumor growth, high hormonal serum levels, and resistance to standard treatments (medical therapy, surgery, or radiotherapy), determining high morbidity and mortality [6]. The prognosis of these tumors strictly depends on their biology, and the search for reliable clinical and pathological markers as predictors of tumor behavior continues.

The need to identify efficient treatments for managing aggressive PitNETs has increasingly emerged. Temozolomide was the first chemotherapy drug to be used as monotherapy and combined with radiotherapy or 5-fluorouracil, with variable results depending on the type of PitNET and previous treatments [7,8,9,10]. Recently, gene alterations, anomalous expression of specific molecular pathways, and regulatory molecules have become objects of interest in discovering new target therapies thanks to the technological improvements in molecular biology, genetics, and bioinformatics. Different studies have brought new knowledge for future treatment options, laying the foundations for precision or personalized medicine. Emerging therapies such as immune checkpoint inhibitors (ICIs), vascular endothelial growth factor (VEGF) receptor therapy, tyrosine kinase inhibitors (TKIs), mammalian target of rapamycin (mTOR) inhibitors, and peptide receptor radionuclide therapy (PRRT) have been tested on a limited number of patients, with variable results [6,11,12,13].

This study aims to offer an exhaustive state-of-the-art review of the principal pathways involved in aggressive PitNETs, detailing the related potential target medical therapies.

## 2. Material and Method

### Search Strategy and Data Extraction

The present study was realized following the 2020 PRISMA guidelines for systematic reviews [14]. The search strategy was performed on 15 June 2023, by two independent authors (S.S. and L.A.) exploring three different electronic databases (Pubmed, Scopus, and Web of Science) with the data filter 2004–2023. The combination of the following keywords was used: (invasive OR aggressive OR recurrence) AND (pituitary adenoma OR pituitary tumor or PitNET) AND (target therapy OR molecular pathway OR genetic pathway OR marker).

The papers included met the following inclusion criteria: (1) studies written in English; (2) publications from 1 January 2004 (year of publication of the WHO Classification of Tumors: Pathology and Genetics of Tumors of Endocrine Organs [15]) to 15 June 2023; (3) original studies on aggressive/invasive PitNETs, (4) papers on pathways, regulatory molecules, and target genes for which drugs are currently available. Among the exclusion criteria, we considered (1) articles written in languages other than English, (2) case reports, and (3) articles that did not add new data (guidelines, letters to the editor, reviews, and editorials).

After removing duplicates, the papers pertinent to the study were initially selected by two authors (S.S. and L.A.) by analyzing the title and then the abstract. At each stage, the consultation of two other authors (S.C. and F.D.) resolved the disagreements between the two reviewers (S.S. and L.A.). The papers were collected and divided for the full-text phase based on the several pathways, regulators, or molecules described, distinguishing between in vitro and in vivo results. For the quality assessment, the Critical Appraisal Skills Programme [16] (https://casp-uk.net/casp-tools-checklists/) and the Joanna Briggs Institute (JBI) (accessed on 29 June 2023) were used to conduct the systematic review [17].

## 3. Results

**Figure 1:**
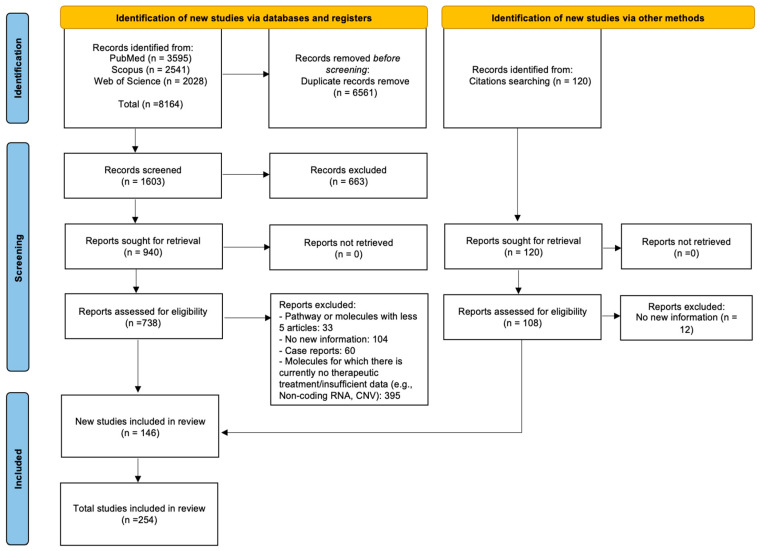
The Preferred Reporting Items for Systematic Reviews and Meta-Analyses (PRISMA) diagram of the article search according to the guidelines [14].

The search of the three electronic databases (Pubmed, Scopus, and Web of Science) yielded 8164 articles. After removing 6561 duplicates, 1603 papers were screened first based on the title and then on the abstract, resulting in 738 studies eligible for the full-text phase. Moreover, 108 studies were manually added through the analysis of the references. A total of 254 articles were included in the literature review. Figure 1 summarizes each step of the literature search according to the 2020 PRISMA guidelines [14].

## 4. Discussion

Table 1 summarizes the current targeted therapies available.

### 4.1. Tumor Microenvironment

**Figure 2:**
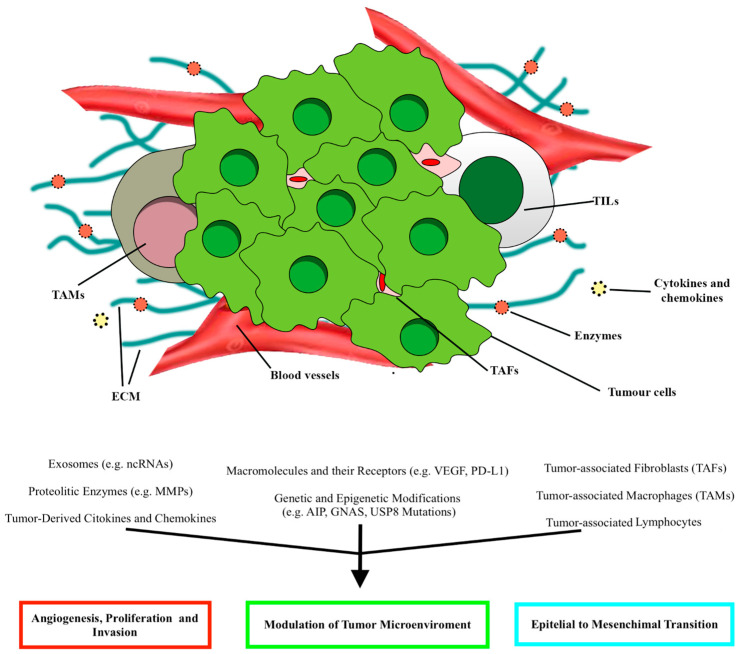
Schematic illustration of the main elements constituting the tumor microenvironment (TME) in PitNETs and the relative effects, according to Marques and Korbonits [20]. ECM, extracellular matrix; TAFs, tumor-associated fibroblasts; TALs, tumor-associated lymphocytes; TAMs, tumor-associated macrophages.

The tumor microenvironment (TME) is characterized by the interplay between the host and tumor cells. TME comprises non-tumor cells, vascularization, extracellular matrix, cytokines, and enzymes (Figure 2) [11,18]. The immune infiltration is eterogenous among the different subtypes of PitNET. As the TME modulates tumorigenic mechanisms such as cell proliferation, invasiveness, and angiogenesis, it may help explain PitNETs’ heterogeneous behavior [11,19]. The TME study in PitNETs has been expanding over the last few years, leading to the identification of new biomarkers for prognostic stratification and the development of novel therapies.

Below is an overview of all the TME factors that play a role in PitNETs’ behavior (Figure 2) and the related target therapies.

#### 4.1.1. Macrophages

Macrophages, distinguished in M1 and M2 subpopulations, are immune cells infiltrating mainly sparsely granulated somatotropinomas, null-cell adenomas, and gonadotropinomas.

While M1-macrophages are characteristic of normal pituitary, the predominant population in PitNETs are M2-macrophages, characterized by pro-tumorigenic effects and associated with worse outcomes [19]. They showed a pro-angiogenic role in estrogen-induced rat prolactinomas and a tumorigenic function in diethylstilbestrol-induced rat prolactinomas [18,20].

In somatotropinomas, higher amounts of tumor-infiltrating CD68+ macrophages, widely present in sparsely granulated forms, correlated positively with tumor volume and higher expression of Ki-67 and MMPs, cavernous sinus invasion [21]. High amounts of macrophages were also revealed in AIP mutation-positive somatotrophinomas [20].

#### 4.1.2. Lymphocytes

The role of lymphocytic infiltrate in pituitary tumors has been described, including CD3+, CD45+, cytotoxic CD8+ T cells, and CD4+ T helper, in particular in functioning pituitary tumors, and, to a lesser extent, also B cells, neutrophils, and mast cells [22]. Some recent studies reported significant infiltrates of natural killer (NK) cells and FOXP3+ T cells in the microenvironment of PIT1-positive pituitary tumors [23].

In Heshmati et al., the lymphocytic infiltrate was found in 2.9% (40 patients) out of a sample of 1400 adenomas, mainly in PRL (19 patients) and multihormonal (8 patients) adenomas, while it was not documented in gonadotroph and TSH PitNETs [24].

Different lymphocyte subtypes may have potentially anti- or pro-tumor effects. In pituitary tumors with a Ki-67 ≥ 3%, lower CD8:CD4 and CD8:FOXP3 T cell ratios and higher levels of macrophages, T helper, FOXP3+, and B cells were reported [20]. Invasiveness has been associated with a higher CD8+ T cell count and a higher FOXP3:CD8 cell ratio, especially in non-functioning pituitary adenomas (NF-PitNETs) [25]. Huang et al. demonstrated high expression of IL-10, low CD56+ and CD28+ cell infiltration in tumor samples, a low percentage of CD3− CD56+ NK cells, high CD3+ CD8+ CD28− T cells, and IL-10 in peripheral blood in patients with invasive NF-PitNETs [26]. Furthermore, Wang et al. identified a CD8+ cell lymphocytic infiltrate in 66 of 191 PA patients [27].

Conversely, in the somatotropinomas, CS invasion was not associated with high infiltration of CD8+ T cells, and CD68+, CD4+, and CD45+ cell infiltration were related to endothelial markers [20]. In another study, an association between a high level of VEGFA and CD163 (an M2-macrophage marker) was also reported [28]. Moldovan et al. demonstrated the potential prognostic value of CD44, documenting its significant expression in invasive and recurrent PitNETs [29]. Also, Qu et al. investigated the potential role of CD147 as a predictive biomarker, speculating a pivotal role in the development and progression of invasive PitNETs [30].

#### 4.1.3. Stromal Cells

Stromal cells include tumor-associated fibroblasts, myoepithelial cells, and pericytes and can enhance tumor cell proliferation and invasiveness. Three subtypes of tumor-associated fibroblasts have been described: antigen-presenting fibroblasts, inflammatory fibroblasts, and myofibroblasts [23]. Tumor-associated fibroblasts seem to play a crucial role in the progression of PIT1-positive and corticotropic tumors. In invasive PitNETs, fibroblasts are characterized by higher expression of both α-smooth muscle actin (α-SMA) and VEGF, positively influencing the proliferation of GH3 pituitary tumor cells [31]. According to Azorin et al., the invasion mode of GH3 cells could be conditioned by the collagen subtype: the presence of collagen type IV may favor an invasive phenotype; on the other hand, collagen types I and III could reduce the invasion rate [32].

A potential association between IL-6 and CCL2, secreted by tumor-associated fibroblasts, and high Ki-67 levels was found, speculating how tumor proliferation in PitNETs may be influenced by fibroblasts [20,33].

In a recent study on silent corticotrophinomas, several alterations of the actin cytoskeleton, organization of secretory vesicles, and expression of genes related to proopiomelanocortin were identified not only in tumor cells but also in stromal cells, providing novel insights into the invasiveness of silent corticotroph PitNETs [34].

#### 4.1.4. Folliculo-Stellate Cells

Follicle-stellate cells are identified in most PitNETs, and through their ability to release growth factors and cytokines, they maintain a balance between the different cell types and perform immune functions [35]. Recently, an association between increased S100B+ folliculo-stellate cells and lower tumor proliferation was positively related to the expression of estrogen receptor-α and FSH in gonadotropinomas [36]. Higher growth hormone levels were secreted in another study on somatotropinomas with scattered folliculo-stellate cells [37].

#### 4.1.5. Cytokines, Chemokines, and Growth Factors

Cytokines, growth factors, and chemokines, produced by tumor cells and those surrounding the tumor, can trigger cellular defense mechanisms and regulate tumor progression. Cytokines and growth factors, such as CXCL12, CCL5, CCL17, IL-8, IL-6, IL-1, IL-2, IL-17, tumor necrosis factor-α, and vascular endothelial growth factor (VEGF), can affect tumorigenic mechanisms in pituitary neoplasms [20]. Chemokines, such as CXCL8 (or interleukin (IL)-8), CCL2, CCL3, and CCL4, are secreted by pituitary tumors and non-tumor cells, such as macrophages, lymphocytes, or fibroblasts, and modulate the microenvironment composition [20,25].

In a recent study, macrophage-derived CCL17 drove tumor invasion by acting on the CCL17/CCR4/mTORC1 pathway, suggesting that chemokines secreted by non-tumor cells may positively favor the invasiveness of PitNETs. Furthermore, a greater expression of CCL17 was identified in large and invasive tumors, hypothesizing a potential association [38].

The increased expression of immune checkpoints, such as the programmed death ligand-1 (PD-L1) and the cytotoxic T-lymphocyte-associated protein 4 (CTLA-4) ligands CD80 and CD86, has been associated with aggressiveness in proliferative pituitary tumors [20]. A study highlighted the potential angiogenic effect of CCL2 released by pituitary tumors, finding larger vessels in pituitary tumors secreting higher levels of CCL2 [20]. According to recent data, tumor-infiltrating macrophages have been mostly described in those PitNETs with hypersecretion of CCL2, CCL3, CCL4, and IL-8 [25]. At the same time, CD8+ T cell infiltrates are found in tumors with high levels of CCL2 and CCL4 [25]. Gonadotroph tumor cells polarize macrophages to the M2 phenotype, while somatotroph and gonadotroph tumor cells can recruit macrophages via CCL5 and colony-stimulating factor-1 (CSF-1) [21]. Another study reported that increased CCL17 expression was associated with a higher concentration of M2 macrophages in pituitary tumors. Moreover, higher expression of CCL22, CCL24, and CCL27 may increase the number of B and CD8+ T cells [22].

The pituitary tumors characterized by elevated expression of PD-L1 presented prevalent immune infiltrates of CD4+, CD8+, and FOXP3+ T cells, highlighting the ability of pituitary tumor-infiltrating immune cells to modulate the expression of immune checkpoint regulators [20]. Moreover, increased expression of the aforementioned CD163 was positively associated with higher expression of PD-L1, PD-L2, and lymphocyte activating 3 (LAG3) [20,28]. The increased presence of macrophages, CD4+, CD8+, and CD45+ T cells was related to the expression of V-domain immunoglobulin suppressor of T cell activation (VISTA) and LAG3 [28].

Kim et al. demonstrated that transforming growth factor-β (TGF-β) signaling was down-regulated in invasive NF-PitNETs. Furthermore, the invasive nature of NF-PitNETs appeared to be associated with overexpression of claudin-9 (CLDN9), down-regulation of insulin-like growth factor binding protein 5 (IGFBP5), death-associated protein kinase 1 (DAPK1), and tissue inhibitor of metalloproteinase-3 (TIMP3) [39].

Wang et al. identified PKCθ as a critical enzyme for bone invasion in PitNETs, thanks to the monocyte-osteoclast differentiation achieved through the release of IL-1β [40].

#### 4.1.6. The Role of Immunotherapy in Pituitary Tumors: Response and Outcomes

**Figure 3:**
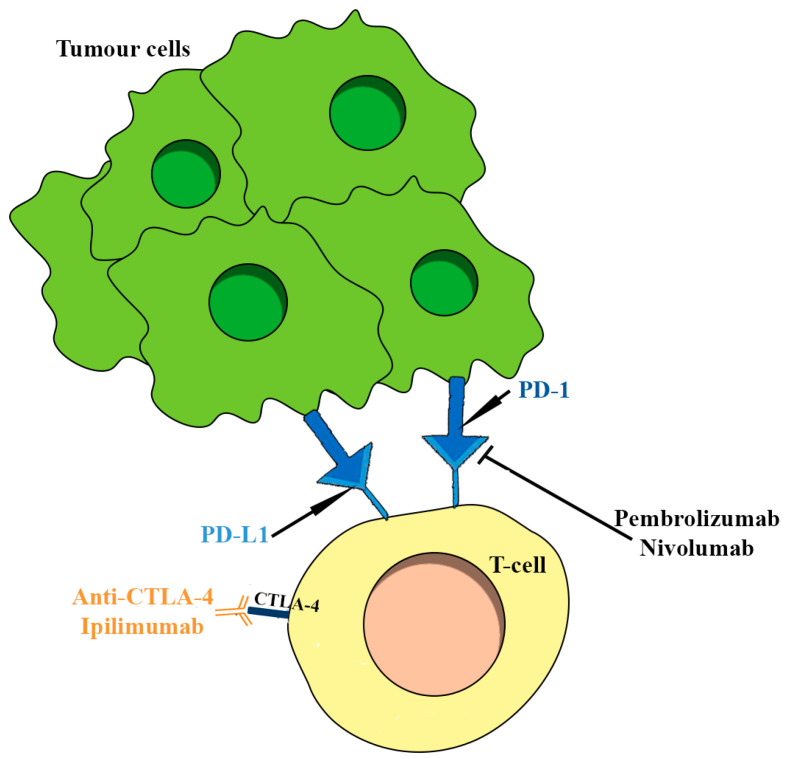
Graphical representation of the relationship between pituitary tumor and T-cell, with emphasis on target therapies, according to Voellger et al. [41].

The efforts of the last decades in understanding TME paved the way for new therapeutic options, such as immune checkpoint inhibitors (ICIs). The growing association between the aggressive nature of PitNETs subgroups unresponsive to conventional treatments and the expression of immune checkpoints has increasingly determined the use of drugs targeting CTLA-4 (ipilimumab) or PD-1/PD-L1 (nivolumab or pembrolizumab) (Figure 3) [41]. Only a few cases of pituitary tumors treated with ICIs have been reported. Using these drugs in single (such as pembrolizumab) or combined formulations (ipilimumab and nivolumab) allowed a partial radiological response to be obtained in five cases of pituitary carcinoma [6,42,43]. In comparison, in two other cases, the disease remained stable [6,42,43]. Maintenance therapy with nivolumab was used in those cases of pituitary carcinoma with an initial response to ipilimumab and nivolumab, showing a radiological response. In the case of disease progression, the carcinoma was retreated with ipilimumab and nivolumab without success, while in the others, a radiological response was documented [43]. Progression-free survival (4 to 42 months) was found in three carcinomas treated with pembrolizumab [43].

The latest efforts aimed at determining which patients could benefit from immunotherapy or how to improve/enhance the therapeutic response for each patient, such as the combined effect of checkpoint inhibitors with peptide receptor radionuclide therapy [44], hypothesize that radiation may influence the TME, further sensitizing the pituitary tumor to the therapeutic response of immunotherapy.

Concerning the individual components of the TME, a tumor-infiltrating immune profile has been associated with poorer outcomes and recurrence in PitNETs [6]. Moreover, the identification of CD8+ T cells could predict the response to first-generation somatostatin analogs, regardless of tumor characteristics (hormone levels, size, and invasion) and patient age, as demonstrated by Chiloiro et al., where somatotropinomas with high levels of CD8+ and CD138+ lymphocytes responded to first-generation somatostatin analogs [45]. According to a recent study, anti-PD-L1 target therapy could act on those subgroups of PitNETs characterized by elevated Ki-67 and p53 values, with a higher percentage of tumor-infiltrating CD68+ macrophages and CD8+ TILs [46]. However, it is not possible to exclude a response even in those cases with negative PD-L1 staining and low infiltration of CD8+ T cells, such as in corticotropinomas [47]. It should be emphasized that recurrent PitNETs are characterized by a higher density of CD163+ and CD204+ macrophages, higher ratios between CD163:Iba1 and CD204/Iba1 macrophages, elevated expression of CSF-1 [48], as well as CTLA-4 ligands (CD80 and CD86) [49]. Therapies that block the CSF-1/CSF-1R axis and that, in general, chemokine-targeted therapies (antiCXCL12 or anti-CCL2) not only have the effect of acting directly on tumor growth and propagation but also target the TME formed by immune cells [21].

In addition, traditional therapies can influence TME. Somatostatin and its analogs are characterized not only by their anti-tumor action but also by the possibility of influencing the pituitary tumor microenvironment, affecting fibroblasts, and inhibiting the release of cytokines, growth factors, and VEGF from pituitary tumor cells [20,33,50,51]. The immunomodulation of dopamine with DAs, thanks to the presence of receptors on endothelial cells, macrophages, and lymphocytes, has also been described, highlighting the inhibitory effect of cytokine and growth factor secretion, blocking chemotaxis, and inducing apoptosis, especially during the management of prolactinomas and somatotropinomas [52,53,54]. Chauvet et al. describe the dual effect of bromocriptine on blocking tumor growth and normalizing blood vessels in a mouse model of prolactinoma [55]. Recently, both anti-angiogenic drugs, such as bevacizumab, and tyrosine kinase inhibitors, such as sunitinib, axitinib, lapatinib, or imatinib, have been identified as potential drugs for the treatment of aggressive pituitary tumors resistant to conventional treatments [18,20].

Studies trying to identify alternative targets have been flourishing in recent years. Recent research has identified HSPB1, which is involved in tumor progression by modulating the immune response, as a potential target in invasive pituitary tumors, as inhibitors of HSPB1 expression are currently available [56].

Further understanding of the PitNETs TME might lead to additional prognostic and therapeutic markers for personalized medicine.

### 4.2. PI3K/Akt/mTOR and RAS/MEK/ERK Pathways

The PI3K/Akt/mTOR and RAS/MEK/ERK pathways are fundamental for cell survival, proliferation, migration, regulation of protein, lipid, and nucleic acid metabolism, senescence, and autophagy. They are triggered by the bond between extracellular ligands and receptor-linked tyrosine kinases (RTK); this bond triggers a cascade of phosphorylation-type reactions that ultimately activate or deactivate various substrates, including transcription factors. This bond, for example, can activate the phosphatidyl-inositol-3-kinase (PI3K), which in turn stimulates the formation of mTOR Complex 1 (mTORC1) and 2 (mTORC2), two protein complexes through which mTOR can operate its effects. Growth factors binding their RTKs also trigger a protein of the Ras family (like H-Ras or K-Ras), which prompts a cascade of sequential phosphorylation-type events that activate RAF, then MEK1/2, then ERK1/2. These kinases interact with various enzymes and transcription factors and also converge on the mTOR pathway. The PI3K/Akt/mTOR and RAF/MEK/ERK pathways are involved in many human neoplasms [57,58,59].

Several studies have analyzed the PI3K/Akt/mTOR and RAS/MEK/ERK pathways in PitNETs and the impact of anti-mTOR target therapy. However, most have been performed in vitro or with mouse models, so further clinical research is needed.

EGFR signaling, which involves the mTOR pathway, is expressed in these tumors, and it is associated with tumor proliferation, invasive behavior, lower total resection, and epithelial-to-mesenchymal transition [60,61,62]; this latter is also mediated by the ADAM12 metalloprotease [62]. IGF-1 signaling also exerts a mitogenic effect through the PI3K/mTOR/Akt pathway [63,64].

Expressing platelet-derived growth factor (PDGF) and its receptor by folliculostellate cells stimulates pituitary cell proliferation through the PI3K/mTOR/Akt pathway [65].

Mutation of the proto-oncogene PI3KCA (which encodes a subunit of PI3K) was found in 2.3% to 12.1% of tumor series, while amplification of the same gene was documented in 21.2% to 28% of cases [66,67]. Mutations of PTEN do not seem to occur frequently [68], but it is downregulated in PitNETs [69]. In its phosphorylated form, Akt is more expressed in PitNETs than in normal pituitary tissue [68,70,71,72,73,74,75], especially in recurrent tumors [71].

The mTOR molecule was shown to be expressed in PitNET cells [76] alongside two mTOR-related proteins, RAPTOR (part of mTORC1) and RICTOR (part of mTORC2) [69], with RAPTOR expression being associated with CS invasion. Unlike normal pituitary tissue, downstream effectors of mTOR, like phospho-S6 protein and phospho-4EBP1 protein, are increased in PitNETs [74,77]. DEPTOR, a down-regulator of mTOR, is underexpressed in PitNETs [78].

The RAF/RAS/MEK/ERK pathway, and especially the ERK molecule, is also overexpressed in PitNETs [62,63,70,71,76,77,79,80,81,82] and was shown to be regulated by the PI3K/Akt/mTOR pathway [82]. ERK also mediates the signaling of different growth factors, like EGFR [60] and IGF-1 [64], in pituitary tissue. The transcript of BRAF is overexpressed in pituitary adenomas compared to normal pituitary [83], and the BRAFV600E mutation was found in 16.5% of corticotroph adenomas [84]. Mutations of RAS were found in 7% of invasive PitNETs [67] and were reported in three metastases of PitNETs but not in their respective primary neoplasms [85].

In summary, current evidence points to overexpression and hyperactivation of crucial molecules of the PI3K/Akt/mTOR and RAF/MEK/ERK pathways in PitNETs.

#### Target Therapy against the PI3K/Akt/mTOR and Ras/Raf/MEK/ERK Pathways

**Figure 4:**
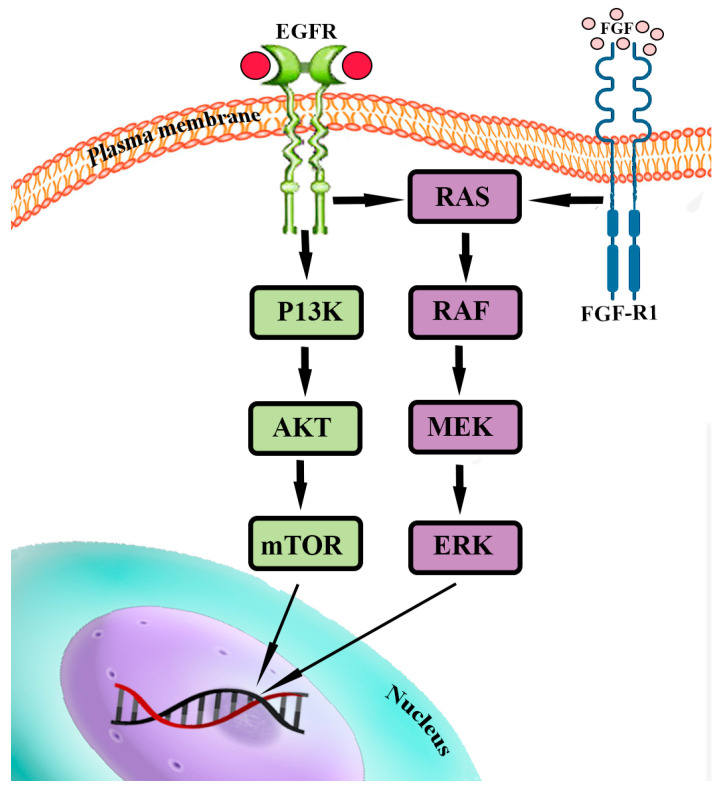
Graphical representation of PI3K/Akt/mTOR and Ras/Raf/MEK/ERK pathways according to Voellger et al. [41].

A few drugs targeting the PI3K/Akt/mTOR and the Ras/Raf/MEK/ERK pathways have shown efficacy in vitro and in mouse models against PitNET cells (Figure 4) [41].

Rapamycin (also known as Sirolimus) and its analog Everolimus (also known as RAD001) inhibit mTOR directly by binding the FKBP12 protein, forming a complex that interacts with the mTOR molecule and prevents it from forming mTORC1 and mTORC2. These drugs can reduce the number of viable PitNET cells, their proliferation, and the phosphorylation of downstream mTOR effectors [73,75,86,87,88], lower prolactin secretion, decrease mTOR phosphorylation, enhance the radiotherapy response, and block IGF-I proliferative and anti-apoptotic effects [86,88]. The anti-proliferative effects of Everolimus are enhanced by the co-treatment with Pasireotide [88], and a similar effect was observed for Rapamycin with Octreotide [89]. Indeed, the anti-proliferative effect of Octreotide seems to be also mediated by components downstream of PI3K, including reduced phosphorylation of PDK1 and Akt, the induction of tumor suppressor gene Zac1 (also involved in the PI3K pathway), and increased phosphorylation of IRS1, a molecule that provides a negative feedback effect on the mTOR pathway [89,90].

Inhibitors of PI3K, like the pan-PI3K inhibitor NVP-BKM120 (Buparlisib) and the specific PI3K-alpha inhibitor NVP-BYL719 (Alpelisib), have shown a dose-dependent inhibition of cell viability of PitNETs and display a synergistic effect when combined with Everolimus [87]. Inhibition of PI3K with the chemical compound LY294002 reduces PitNET cell growth, increases the pro-apoptotic activity of Bcl2-associated death promoter, decreases the anti-apoptotic effect of IGF-1, and decreases phosphorylation of PI3K and Akt [64,74,91,92,93].

Dual PI3K-mTOR inhibitors like NVP-BEZ235 (Dactolisib) seem more effective than Everolimus in reducing the cell viability of PitNETs [94]. NVP-BEZ235 treatment decreases Akt and S6 phosphorylation and triggers apoptosis [94,95]. Another dual inhibitor, XL765 (Voxtalisib), enhances the effects of temozolomide against PitNET cells [95].

Gefitinib, an anti-EGFR tyrosine kinase inhibitor, reverses the epithelial-to-mesenchymal phenotype, decreases invasiveness, and reduces the proliferation of PitNETs [62]. In another study, Gefitinib resulted in tumor shrinkage and a reduction in peripheral hormone levels by around 30% in a mouse model. Gefitinib treatment in mice decreased ERK1/2 phosphorylation, followed by downregulation of tumor prolactin mRNA [60].

Among other lesser-known drugs, the Akt inhibitor MK-2206 was shown to reduce the phosphorylation of Akt. The HIV protease inhibitor Nelfinavir radiosensitizes PA cell lines in vitro, and the underlying mechanism seems to involve the mTOR pathway [96].

Corticocotroph PitNET cells harboring the BRAFV600E mutation undergo a more significant reduction in hormone secretion when treated with BRAF inhibitor Vemurafenib, compared to tumor cells with wild-type BRAF [84].

In summary, drugs targeting the PI3K/Akt/mTOR pathway seem capable of interfering with PitNET growth, survival, and hormone secretion and enhancing the effects of other therapeutic strategies like radiotherapy, somatostatin analogs, and temozolomide. Still, clinical studies are required to study their effectiveness further.

### 4.3. Receptors

#### 4.3.1. Somatostatin and Dopamine Receptors

Somatostatin receptors (SSTR) and dopamine receptors (DRD) represent a staple in PitNETs therapy, and their agonists are commonly used in clinical practice, especially in PIT-1-positive tumors (GH and PRL-positive subtypes), except corticotrophs and gonadotrophs. In particular, in Cushing’s patients, SSTR5, SSTR2, and SSTR3 are predominantly expressed, representing three of the four target receptors of pasireotide [97,98]. However, we must still define their relationship with PitNET pathophysiology.

The main subtypes expressed by PitNETs are SSTR2 and SSTR5, and increasing evidence shows that when SSTR2 is less represented, PitNETs show more aggressive behavior [99,100]. Brzana et al. [101] correlated SSTR2 expression with granulation pattern in GH-secreting tumors: scarcely-granulated tumors showed a lower expression of SSTR2, and densely-granulated tumors with a significant expression of SSTR2 were more likely to respond to somatostatin receptor ligands, as confirmed by Venegas-Moreno et al. [102]. From a prognostic point of view, low/absent cytoplasmic SSTR2 expression is correlated with recurrence rate, reintervention probability, and poor response to somatostatin receptor ligands (SRLs) therapy in acromegaly [98,103,104].

**Figure 5:**
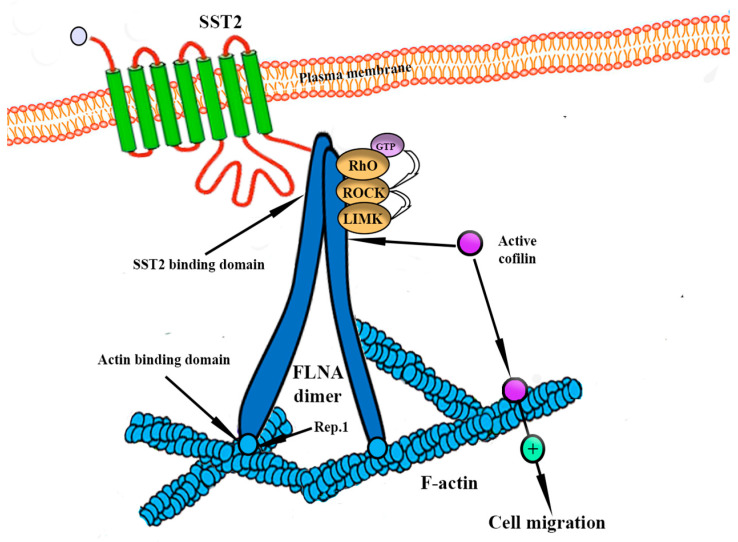
A schematic illustration of the molecular mechanism among SST2, FLNA, and the cofilin pathway in GH-secreting pituitary tumor cells and its consequences on cytoskeleton remodeling and cell migration, according to Peverelli et al. [103].

A novel pathway has been described by Peverelli et al. (Figure 5) that might explain SSTR2 anti-neoplastic activity and its role in PitNETs pathophysiology via the activation of the Rhoa/ROCK pathway and the consequent cofilin phosphorylation; this way, cofilin is unable to bind to actin, and SSTR2 activation inhibits cytoskeleton remodeling and cell migration [103]. The exact mechanism for the DRD2 isoform was described by Peverelli et al., who also correlated low cofilin phosphorylation with invasion in aggressive PitNETs [104]. New molecules have been proposed, such as BIM23120 (a selective SSTR2 agonist) and BIM53097 (a selective DRD2 agonist), showing promising results in reducing somatotroph cell migration and proliferation and inducing apoptosis. Nevertheless, it has to be noted that there are patients who do not respond to SRL therapy: although pasireotide reduced ACTH and cortisol levels in Cushing’s patients in the phase II trial conducted by Boscaro et al. [105], ACTH-secreting tumors usually become unresponsive to SRL-agonists, and this phenomenon might be explained by the downregulation of SSTR2 by cortisol [106]; many studies showed a positive correlation between SSTR expression and responsiveness to SRL-therapy [102,107].

SSTR5 is highly expressed in NF-, ACTH-, and GH-PitNETs and correlates with recurrence [98]. When analyzed in association with DRDs, it has been shown to form chimeric receptors, particularly with DRD2 [108,109], and the chimeric receptor SSTR5/DRD2 has been linked with inferior dimension and grade [109]. Its targeting via BIM23A760 (a selective agonist) reduced GH and PRL production in vitro; in GH tumors, it reduced cell viability and proliferation and stimulated apoptosis [108]. Nevertheless, some specimens did not respond to BIM23A760 administration. It appeared to be associated with SST5TMD4, a truncated isoform of SSTR5 highly represented in PitNETs (particularly GH-positive), positively correlated with sphenoid and CS invasion, and implied in SRL resistance; it is often co-expressed with SSTR2, and Luque et al. demonstrated that the two proteins hetero-dimerize, and when that happens, SSTR2 membranous expression is sensibly reduced. At the same time, the intracellular is enhanced [107]. Accordingly, when SST5TMD4 is present, BIM23A760 induces stimulatory rather than inhibitory responses [108]. The presented evidence highlights the therapeutic effects of SSTRs. It pinpoints new molecules that might, in the future, be adopted in PitNETs treatment (e.g., BIM23A760, BIM23120) while also adding potential markers of response to medical therapy (SST5TMD4) and of aggressivity (cofilin).

Other SSTRs were less investigated, given their lower representation. The data above have revived interest in these receptor subtypes, and there is evidence that SSTR1 might represent an indicator for response to medical treatment and a prognostic factor: its overexpression has been described in recurrent ACTH-secreting tumors [98], and low levels are linked with remission after first surgery [107]. However, Venegas-Moreano et al. [102] described a negative correlation between SSTR1 and tumor dimension and a positive connection with responsiveness to medical treatment in acromegalic patients, suggesting a multimodal behavior of SSTR1 when it comes to different PitNET subtypes (similar to what happens with SSTR2 and ACTH-PitNETs as mentioned above). Nevertheless, there are enough data to make SSTR1 a plausible candidate for future therapies, but more studies are needed to investigate its role in different PitNET categories.

As for SSTR3, Lee et al. suggested its anti-neoplastic role is due to its implication in the MAPK pathway: SSTR3 activated tyr-phosphatases, ultimately inhibiting MAPK and consequently activating p53 and the caspases, promoting apoptosis [110]. SSTR3 is highly expressed in SF-1 positive PitNETs, even recurring ones [110], while it is reduced in recurring NF PitNETs [98].

Understanding SSTRs and DRDs is emerging as a crucial point in the pathophysiology and therapy of PitNETs; further studies are needed to clarify their exact mechanism and involvement in different PitNET subtypes so that specific treatment can be tailored to the molecular characterization of the tumor.

#### 4.3.2. Peptide Receptor Radionuclide Therapy

Peptide receptor radionuclide therapy (PRRT) is an innovative therapeutic option traditionally used as a second-line treatment for advanced (metastatic or inoperable) neuroendocrine tumors (NETs), characterized by the expression of the SSTRs, especially the SSTR2 [111,112]. The applicability of such treatment is closely related to the expression of somatostatin receptors, which can be evaluated through functional imaging such as 68Ga-DOTA peptide PET/CT, octreoscan, 111In-octreotide-scintigraphy, or 99m-EDDA-HYNIC-tyr3-octreotide scintigraphy [10,113,114,115,116,117,118,119,120,121,122,123,124]. Several types of radionuclides exist (111In, 177Lu, 90Y, and 68Ga), combined with chelators (DTPA or DOTA) and somatostatin receptor ligands (TATE, Octreotide, or TOC). Recently, it was also introduced for managing aggressive PitNETs or carcinoma in cases of the failure of conventional therapeutic options. The data currently available in the literature are still limited to clinical cases [10,113,114,115,116,117,118,119,120,121,122,123,124], demonstrating disease progression in most cases. Only in a few patients was a partial response documented by a reduction in the tumor volume and hormone levels [10,113,114,115,116,117,118,119,120,121,122,123,124]. The PRRT outcome was strictly related to a low Ki-67 index, slowly progressive disease, and not using temozolomide as prior medical treatment [115,121]. However, no differences in response to treatment are currently found among the different radionuclides used, age, or gender [115]. Although the treatment is commonly well tolerated, undesirable effects have been reported, such as suppression of blood cells in a patient affected by GH-PitNET and progressive facial pain in ACTH-PitNET with subsequent treatment discontinuation [119,123].

Among the possible future perspectives, it will be necessary to define the role of radiopeptides in combination with chemotherapies or immunotherapy and identify different radiopeptides for diagnosis and treatment. Some promising results have been obtained through synaptic vesicle glycoprotein (SV2A) expression in PET imaging for neuroendocrine differentiation in prostate cancer [125].

#### 4.3.3. Transforming Growth Factor Receptor

Transforming growth factor beta (TGF-β) signaling is related to numerous biological processes involved with cell proliferation, differentiation, apoptosis, and EMT. Its action is mediated by TGF-β receptor complexes (TGF-β RI and RII) that phosphorylate Smad2 and Smad3, which in turn form trimers with Smad4 and translocate to the nucleus, regulating the expression of genes controlling the cell cycle [126]. TGF-β has been investigated in different types of cancer, and it can act as either a suppressor or an inhibitor of tumor development, depending on the tumor and stage [127]. Given its intricate relationship with tumorigenesis, new data are being implemented to uncover its role in PitNETs.

Some authors have described that TGF-β is elevated in aggressive PRL-secreting tumors, both locally [128] and in the serum (and serum TGF-β is directly correlated with tumor dimensions and aggressivity) [129]. Jiang et al. correlated TGF-β levels with microvascular density in the neoplastic tissue [128]. Similar evidence was found by Dallago et al., who directly related TGF-β with tumor extension [130], and by Zhu et al., who demonstrated that NF-PitNETs recurrence showed higher expression of TGF-β [131]. These data suggest that TGF-β could represent a viable marker for PitNETs aggression and recurrence. Moreover, Duan et al. tested Tioglitazione (“TGZ”, an agonist of PPAR-c) as a potential therapeutic agent. They demonstrated that somatotroph cellular lines exposed to increasing concentrations of TGZ reduced the expression of TGF-β [132].

On the contrary, when analyzing the TGF-β pathway, Ying-Hao et al. reported an inverse association between TGF-β RII expression and invasiveness, thus documenting it as a tumor suppressor. No significant difference was highlighted for TGF-β RI [133]. The same conclusion was drawn by Petiti et al. [134], who also demonstrated that when incubated with trastuzumab, PitNET cell proliferation was inhibited via an enhanced expression of Smad2 and Smad3. This suggests that the TGF-β pathway is far more complex than previously believed, including the EGFR/Erk pathway (which, when activated, reduces Smad2 and Smad3 phosphorylation). This evidence strongly supports the possible implementation of anti-EGFR drugs in PitNETs treatment and opens the way for potential therapeutic implications of targeting the TGF-β mediator. Smad2 and Smad3 are linked with invasiveness in NF-PitNETs [135].

These data suggest a multimodal TGF-β role in PitNETs pathophysiology that intertwines with other relevant pathways in neoplastic evolution (e.g., EGFR/Erk). Further studies are needed to explore the relationship between TGF-β (and its mediators) and different subtypes and grades of PitNETs. Still, researchers are investigating possible molecules for targeting the pathway that might be implemented in PitNETs medical treatment soon.

#### 4.3.4. Fibroblast Growth Factor Receptors

Fibroblast growth factor receptors (FGFR) are tyrosin-kinase receptors, and their activation mediates cell proliferation, migration, and apoptosis [136]. The involved pathways include MAPK and phosphatidylinositol-3-kinase. Ptd-FGFR4 is the N-terminally truncated isoform of FGFR4, characterized by cytoplasmic localization and constitutive phosphorylation [137]. Analyzed against common biomarkers of aggression and invasiveness, ptd-FGFR4 showed a direct correlation with Ki-67 [138]. Accordingly, Brito et al. documented that all patients with recurrent Cushing’s disease had high levels of FGFR4 mRNA expression [139]. Morita et al. demonstrated that ptd-FGFR4 mRNA levels were directly associated with GH-secreting tumor invasiveness [140]. The same evidence was observed by Ezzat et al. [141]: in their series, ptd-FGFR4 was linked to aggressivity and cytoplasmic N-cadherin levels that, in turn, correlated with invasiveness. They also tested PD173074 (an inhibitor of ptd-FGFR4) in GH4 pituitary cells. They observed a reduction in cytoplasmic concentration of N-cadherin, invasion of adjacent structures, and neoangiogenesis versus better inter-cellular adhesion and inferior Rb protein expression, a well-known oncogene. Ramírez et al. [100] observed a high expression of FGFR4 by NF-PitNETs but did not observe a significant relation to tumor size or aggressivity.

FGFRs are increasingly investigated as a marker of aggression and a potential therapeutic target.

#### 4.3.5. Folate Receptor

Folate receptors (FR) are glycosylphosphatidylinositol-anchored membrane proteins involved in the absorption of folic acid, essential for cell proliferation. Among the three human isoforms (α, β, and γ), FRα is overexpressed in some forms of cancer, such as ovarian and cervical carcinomas, and, although less commonly, in lung and breast cancers. At the same time, it is poorly expressed in normal tissues [142]. FRα is strongly upregulated in NF-PitNETs but is absent or downregulated in functioning PitNETs or normal pituitary glands [143,144,145,146]. Moreover, FRα was associated with the Ki67 labeling index and tumor dimension in NF-PitNETs [143,145,147,148]. In the mouse gonadotroph cell line αT3-1, FRα was documented to promote cell proliferation, influencing the NOTCH pathway [149].

In a clinical study on 56 NF-PitNETs, a pre-operative SPECT/CT image after 99mTc-EC20-folate administration documented a sensitivity of 81% and a specificity of 83%, highlighting the possibility of an appropriate selection of patients who could take advantage of this treatment [150]. Liu et al. documented the anti-proliferative and pro-apoptotic effects of FRα-targeted liposomes loaded with doxorubicin (F-L-DOX) in human primary PA cell lines [151]. Moreover, F-L-DOX had an anti-invasive effect by blocking MMP2 and MMP9 release. Recently, the introduction of boron-10-containing carbon nanoparticles targeting FR, an experimental radiotherapy based on the combination of 10B nuclei and neutrons on the capture of thermal neutrons by boron 10, demonstrated to act selectively on primary cultures of NF-PitNETs, inducing apoptosis and reducing cell viability [148].

#### 4.3.6. Estrogen Modulators

The relationship between estrogen hormones (ES) and their receptors (ESR) plays a crucial role in the pathogenesis of PitNETs. In normal human tissue, ERα (also called ER1, ESR1, and NR3A1) and Erβ (known as ER2, ESR2, and NR3A2) are nuclear receptor isoforms that respond to 17β-estradiol (E2), leading to cell proliferation and differentiation, respectively [152,153]. Among the best-known ERα receptors, ERα66 and its variant ERα36, which are situated in both the cytoplasm and plasma membrane, are mostly found [154]. A dysregulation among hormones, estrogen, and ESR could explain their aggressive and invasive behavior, especially in prolactinomas, where estradiol and estrogens regulate the transcription of the prolactin gene, influencing dopamine synthesis and modulating the mitotic activity [155,156,157].

Studies performed on lactotroph pituitary adenomas highlighted aggressive behavior, especially in the male gender, when the expression of ERα was reduced [158,159,160,161,162,163,164], probably associated also with the expression of genes situated on chromosome X (CTAG2, FGF13, and VEGF) that influence the ER pathway [158]. Recently, Mahboobifard et al. found a decreased expression of ERα66 and ERα36 in PRL-PitNETs compared with normal pituitary tissue. Furthermore, invasiveness was associated with low levels of ERα36 and ERα66, while an increased Ki67 index was related to decreased ERα36 expression. A significant inverse association between ERα66 with dopamine-agonist resistance and tumor size was also documented [165].

The results regarding ER expression in other PitNET subtypes are contradictory. A higher expression of ERα was found in NF-PitNET, although conflicting results were found across the patient age range (50 y.o.> vs. <50 y.o.) [166]. Moreover, Manoranjan et al. identified that null cell adenomas, FSH/LH, GH-, and PRL-PitNETs were characterized by relevant immunohistochemical expression of Erα [167].

In another study on post-menopausal women with invasive prolactinomas, higher levels of P450AROM were found compared with non-invasive tumors, and a high expression of ERβ was significantly related to resistance to bromocriptine [168].

Recently, Xiao et al. demonstrated that in bromocriptine-resistant prolactinomas and MMQ cells, the interruption of the positive feedback between high expression of PRL receptor and ERα through the combination of fulvestrant and bromocriptine activates the JNK-MEK/ERK-p38 MAPK pathway and cyclin D1 downregulation, inducing bromocriptine sensitization [169].

Regarding tumor size, these data are inconclusive: several studies described macroadenomas with higher levels of ERα than microadenomas [169,170]. In contrast, others identified an inverse or absent association between ERα expression and tumor dimension [161,162].

The association between ER and invasiveness is still debated [158,171,172]: Zhou et al. found a significantly higher expression of nuclear ERα staining in invasive NF-PitNETs (especially in females) than non-invasive ones. In contrast, ERβ staining decreased in invasive NF-PitNETs. Other studies found lower ERα mRNA levels in non-invasive prolactinomas but also significantly lower levels of ERα in invasive pituitary tumors [162,164,165,167].

Regarding the prognostic role of estrogen receptors, low ERα levels were significantly associated with a higher reintervention rate and earlier reintervention in male patients [173]. Low ERα expression was also found in recurrent PRL-PitNETs positive for galectin-3, with high mitotic activity, and in males [162].

Several preclinical studies have demonstrated the efficacy of selective estrogen receptor modulators (SERMs) and selective estrogen receptor down-regulators (SERDs) on GH3, AtT20, and TtT/GF cells. Both types of drugs have been shown to reduce survival in PitNET cells [156,174,175,176]. SERMs, such as bazedoxifene, clomifene, and raloxifene, have been shown to reduce cell invasiveness, especially clomifene, promoting apoptosis through caspase 3/7 activation and downregulating the expression of MMP-14 and ADAM12 [156,165,166,167,168,169,170,171,172,173,174,175]. Using bazedoxifene for two years in rats reduced the incidence of PitNETs (males and females) [177]. Moreover, Voellger et al. found that incubation with resveratrol and irradiation (4 Gy) reduced cell viability in GH3 and TtT/GF cells [176]. Fulvestrant, an estrogen receptor antagonist, was effective only on GH3 cells, promoting apoptosis by downregulating the IRE1/XBP1 signaling pathway and inhibiting proliferation and prolactin secretion in GH3 cells. Moreover, size, tumor volume, and PRL levels were reduced in F344 rats [156,158,178,179].

In clinical studies, raloxifene decreased circulating IGF-I levels in eight acromegalic male patients [179]. Combining SERMs with DA made it possible to influence hormone levels and tumor size in managing DA-resistant prolactinomas. The administration of raloxifene combined with DAs in patients with prolactinomas demonstrated a 25.9% reduction in PRL level in 71% of cases and the achievement of normoprolactinaemia in two patients [180]. Combining anastrozole with DA in four male patients (19–38 years) led to decreased prolactin levels and tumor shrinkage [181]. The use of tamoxifen in patients affected by acromegaly led to the reduction in IGF-1 in 82% of cases [182] and the normalization of IGF-I in 47% of cases. Moreover, using fulvestrant could induce apoptosis in vivo [157].

#### 4.3.7. Wnt/β-Catenin and E-Cadherin

The Wnt/β-catenin pathway, also known as the “canonical” Wnt pathway, has been linked to many human diseases, cancerous and non-cancerous [183]. Once activated by the canonical pathway, β-catenin translocates into the nucleus. It induces genes involved with cell proliferation and migration (e.g., c-myc, MMP) [183].

Moreover, β-catenin intervenes in cell-cell adhesion by forming a heterodimer with E-cadherin (which, in turn, inactivates the mitogenic function of β-catenin when complexed together); the E-cadherin/β-catenin anchoring complex is known to maintain epithelial cell differentiation and adhesion, and its dysregulation has been recognized as a promoter of epithelial–mesenchymal transition in many cancers [184].

Many authors described a loss of extracellular E-cadherin expression in PitNETs, and an intranuclear accumulation of E-cadherin was observed [29,185,186]. This was also true when E-cadherin expression was associated with PitNETs’ clinical behavior: reduced extracellular and nuclear expression of E-cadherin was statistically associated with increased dimensions, invasion of adjacent structures, and a more aggressive behavior [187,188,189]. Some authors even showed these alterations to be of prognostic significance, as nuclear expression of E-cadherin was correlated with disease-free survival and a longer time to reintervention in clinically silent FSH/LH-PitNETs [190]. At the same time, membranous downregulation was associated with a higher recurrence rate by Zhou et al. [191].

The evidence suggests pituitary cells lose their normal differentiation and transition to a tumorous phenotype when membranous E-cadherin is downregulated. However, therapeutic options are not available yet. E-cadherin might represent a molecular therapy target in the future.

B-catenin shows a multimodal pattern: when analyzed alone, nuclear accumulation of B-catenin positively correlates with PitNETs aggressivity [192,193]. On the other hand, when E-cadherin is considered, B-catenin downregulation is linked to aggressive behavior and recurrence [191], further suggesting that E-cadherin serves as a proto-oncogene in PitNETs. Temozolomide has effectively reduced B-catenin activation and nuclear translocation, impairing cells vitality and promoting their apoptosis. It also reduced prolactin production in PRL-secreting adenomas in animal models [194]. Further studies are needed to single out new drugs able to interfere with these pathways. Still, temozolomide efficacy points out B-catenin and E-cadherin as viable candidates for molecular target therapy.

#### 4.3.8. Galectin-3

Galectin-3 (Gal-3) is a β-galactoside-binding lectin expressed in various types of cancers and plays a vital role in PitNET cell proliferation [195,196]. An increased Gal-3 expression in PRL− and ACTH-PitNETs has been described, but why and how it is involved in pituitary tumor progression is still unclear. Gal-3 levels further increase in the progression from PRL and ACTH-secreting adenomas to carcinomas; gene methylation plays a role in Gal-3 expression, and RUNX1 and RUNX2 transcription factors seem to target its gene directly, enhancing its expression [195]. Yoshii et al. demonstrated that Gal-3 phosphorylation was necessary to inhibit PitNET cell apoptosis [197]. Righi et al. showed a direct correlation between Gal-3 levels, Ki-67 labeling index, and mitotic index, with a recurrence rate and low progression-free survival [195,198]. The same was described by Bima et al., who also linked Gal-3 expression with a low probability of response to dopamine agonist therapy in PRL-secreting PitNETs [161]. Dai et al. [199] associated Gal-3 with PitNETs invasiveness, tumor dimension, and pre-surgical PRL levels in PRL-secreting tumors, but they went further to demonstrate that in vitro MMQ cells transfected with Gal-3 siRNA showed lower concentrations of MMP2 and MMP9 and inferior migration capabilities, while also increasing the apoptosis rate. In vivo, nude mice transfected with Gal-3 siRNA had less tumor volume and inhibited tumor growth compared to control mice.

Although still obscure, Gal-3’s role in PitNET pathophysiology is unmistakable. Further studies should focus on its role in crucial cellular pathways involved with cell cycles and tumor progression checkpoints. They should also look at possible molecules targeting Gal-3 as a potential therapeutic option in PitNETs.

### 4.4. Matrix MetalloProteinases

MMPs are zinc-containing calcium-dependent endopeptidases paramount in extracellular matrix (ECM) degradation and remodeling by acting on several substrates. The increasing evidence of the importance of ECM remodeling in tumor invasion supports the relevance of those enzymes in promoting PitNET local invasiveness.

MMP-9 has been widely studied as it acts by notably degrading type IV collagen, which represents the main component of the basal membrane and the medial wall of the CS. In 1996, for the first time, Kawamoto et al. observed MMP-9 expression to be significantly increased in invasive PitNETs when compared to non-invasive ones [200]. Liu W. et al. found MMP-2 and MMP-9 expressions were higher in patients with CS invasion, with no correlation between MMP expression and tumor size or hormone secretion [201].

Indeed, MMP-9 expression was found to be higher in invasive prolactinomas [202,203,204,205] and a marker for CS invasion, together with MMP-2. Cathepsin K (CTSK), a protease that degrades type I collagen and ECM, thereby contributing to bone resorption and tumor invasion, has been reported as a potential marker for sphenoid sinus and clivus invasion [204,205]. In a retrospective study performed, including sporadic Cushing’s disease after surgery, Liu et al. found a significantly increased expression of MMP-9 in patients with recurrence [205].

Moreover, they also found that the expression of MMP-9 was strongly associated with the recurrence-free interval, suggesting that patients with high MMP-9 expression may need particularly close clinical and radiologic follow-up after surgery [205]. Gong et al. found increased MMP-9 mRNA expression in the invasive PitNETs [206]. However, several studies also showed no correlation between MMP-2 and MMP-9 expression, as previously reported by other groups [207,208].

Protein kinase C (PKC) is a ubiquitous family of enzymes involved in several cellular pathways, such as mediating cell growth and tumor invasion by activating MMP-9. Hussaini et al. demonstrated increased expression and activity of MMP-9 in invasive NF-PitNETs and PKC-activated HP75 cell lines [207]. PKC activated MMP-9 in a highly cell-type-specific manner. Moreover, some studies found that invasive PitNETs were characterized by point mutations of PKC-α and higher overall PKC activity and expression [209,210]. The effect of several biological agents on MMP-9 activity and expression by either positively or negatively influencing PKC overall activity has been tested in different tumor models. For instance, Phorbol-12-myristate-13-acetate (PMA) treatment of HP75 cells has increased the activity and expression of MMP-9. In contrast, specific or nonspecific PKC inhibitors, such as bisindolylmaleimide (BIM), Go6976, Rottlerin, siRNAs, and MMP-9-neutralizing antibodies, have been found to block it [207].

Moreover, the IL-6/JAK2/STAT3 pathway seems to be involved in the invasiveness of pituitary tumors by increasing the expression of MMP-9 [211]. The clinical relevance of the work by Feng et al. resides in the fact that several biological agents against this pathway are currently available [211].

Despite increasing evidence of the paramount involvement of MMP-9 in determining PitNETs invasiveness, some studies also report no association between MMP-9 expression and tumor invasiveness [212].

MMP-9 is not the only member of the MMP family associated with the aggressiveness and invasiveness of PitNETs. Mao et al. found that MMP-8 was significantly overexpressed in invasive PitNETs, while TIMP-1 was lower; this was also true for relative mRNA expression and serum levels [213]. These results showed that increased MMP-8 and decreased TIMP-1 expressions are closely related to invasive PitNETs [213].

Hui et al. [214] proposed MMP-14 as a pivotal driver of tumor invasion by promoting angiogenesis through VEGF (as previously demonstrated in glioma [215] and breast carcinoma [216]). Contrariwise, Ruskyte et al. failed to demonstrate that epigenetic silencing of either MMP-14 or TGFβ-1 genes may play a role in PitNETs development [217].

In conclusion, after reviewing the literature, targeting MMPs is also a valuable therapeutic approach against pituitary tumor progression, despite the challenges and many unsatisfactory results. Multiple MMP inhibitors have been developed and tested in the last decade, primarily in preclinical trials, and further efforts are needed. Nowadays, epigenetic regulation of MMP expression should also be considered a potential therapeutic strategy [218]. Furthermore, it should be considered that targeting MMPs may also improve the delivery of other chemotherapeutics, enhancing their efficacy and reducing their side effects.

### 4.5. Angiogenesis

Angiogenesis represents a key mechanism in tumor progression, traditionally associated with aggressive clinical behavior, limited response to first-line treatment, and poor quality of life. The data about angiogenesis in PitNETs are still unclear and conflicting: several studies have demonstrated elevated angiogenetic factors associated with abnormal vessel architecture, while others have found lower-level expression than in normal pituitary glands [53,219,220,221,222,223,224]. Low microvessel density has been documented in most PitNETs. Aggressive PitNETs and pituitary carcinomas have the highest level of vascular density [225,226]. As it has been found in other types of tumors, the expression of genes and molecules involved in angiogenesis could be the key to introducing new therapeutic strategies for aggressive and invasive PitNETs.

#### 4.5.1. Vascular Endothelial Growth Factor

**Figure 6:**
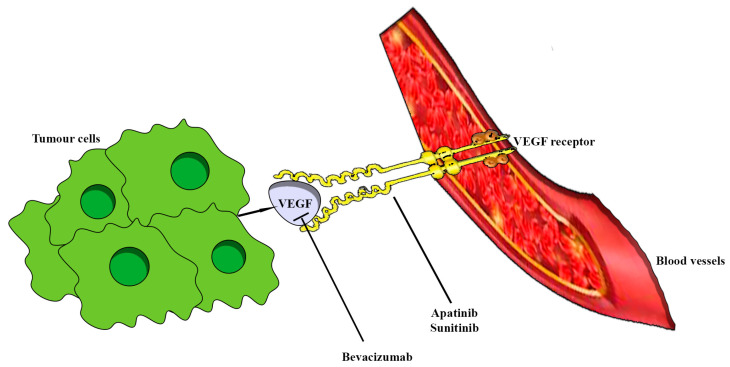
Schematic representation of the VEGF/VEGFr pathway in pituitary tumors (PitNET) according to Dai et al. [229].

Vascular endothelial growth factor (VEGF), especially the subtype VEGF-A, represents a fundamental mediator of vasculogenesis, angiogenesis, vascular permeability, cell survival, and migration [227]. The expression of VEGF is regulated by several factors, such as hypoxia-inducible factor (HIF), epidermal growth factor (EGF), and platelet-derived growth factor (PDGF). In tumorigenesis, the upregulation of VEGF and its receptors is commonly found in most tumors, impacting their invasiveness, the formation of metastasis, and cell survival through the induction of bcl-2 expression [227,228]. Therefore, anti-VEGF therapy has been progressively introduced and approved as a secondary-line therapy (Figure 6) [229]. The available data on VEGF expression for managing aggressive PitNETs are conflicting. Some authors identified overexpression of mRNA VEGF in invasive tumors and carcinomas, especially in the elderly, and NF-PitNETs. At the same time, other studies did not find a significant correlation between VEGF expression and tumor behavior [160,221,230,231,232]. Wang et al. evaluated VEGF expression in 197 PitNETs without finding any association with D2R expression and clinical features (aggressive, recurrence, tumor texture, and bromocriptine application) [233], while Cristina et al. documented a higher concentration and expression of mRNA VEGF in D2R knockout mice than the wild-type group [234]. Moreover, a correlation between IL1a and IL6 and VEGF expressed in 59 cultured adenoma cells was also found [231].

The results from in vitro experiments have shown that the anti-VEGF-A mAb G6-31 inhibited tumor growth and decreased serum prolactin levels, especially in dopamine-agonist-resistant prolactinomas [235]. Zhou et al. experimented with Cabozantinib, an inhibitor of VEGFR2, on RBΔ19 mice, prolonging mean survival in a dose-dependent manner (15 mg/kg and 30 mg/kg) [236]. On prolactinomas, Axitinib, a VEGFR-selective tyrosine kinase inhibitor, stopped tumor progression, decreased vascular density, and, through the combination of bromocriptine, reduced tumor bleeding and normalized the vessel architecture [55]. Moreover, Miyajima et al. showed a significant reduction in the KI-67 labeling index and remodeling of blood vessels in E2-induced prolactinomas during treatment with the anti-VEGF antibody (G6-31) in comparison to E2-induced prolactinomas in F344 rats [237].

Although with encouraging preliminary results, the use of anti-VEGF drugs has been described in a limited number of aggressive PitNETs or carcinomas combined with temozolomide, pasireotide, and radiotherapy. The reported results are heterogeneous and limited by the absence of clinical trials.

It has also been shown that Octreotide also influences the reduction in VEGF expression in GH-PitNETs [238]. The model of diabetic retinopathy may suggest an effect of SSAs on neo-angiogenesis in Pas as well. Amato and co-authors suggested that treatment with Octreotide may reduce the expression and release of VEGF in the retinal vessels of diabetic patients [239]. Therefore, Octreotide may play a role in pituitary tumor angiogenesis, promoting VEGF downregulation.

#### 4.5.2. Endocan

Endocan, also known as endothelial cell-specific molecule-1 or ESM-1, is a soluble chondroitin/dermatan sulfate marker of neoangiogenesis closely related to VEGF and FGF expression. Its role in physiological processes is well known and has an essential effect on inflammation and tumor pathogenesis [240]. In the normal pituitary gland, only a few endocrine cells expressed endocan, while in pituitary tumors, the data about its expression are inconsistent [219,241,242,243].

Cornelius et al. [242] found endocan immunoreactivity in endothelial cells, Miao et al. [241] in tumor cells, and rarely in endothelial cells, while Wang et al. [243] documented prevalent expression in both vascular endothelial and tumoral tissue.

Moreover, Cornelius et al. found an association between endothelial endocan immunoreactivity (IR), recurrence, p53, mitosis count, and greater mean vessel areas [242]. A positive correlation between endocan expression, Knosp grade, and tumor size was confirmed in most studies, although ESM-1 expression was found in tumor or endothelial cells [241,242,243,244].

Even today, the role of Endocan in target therapy is still to be defined, especially for pituitary pathology. Studies on other tumors have shown that it is possible to block endocan expression by acting on the VEGF pathway through tyrosine kinase inhibitors or VEGF antibodies [245]. Recently, monoclonal antibodies, direct synthetic peptides, and endocan silencing have been developed, promoting apoptosis, tumor volume reduction, and cell migration in several tumors [246,247]. In pituitary pathology, an elevated expression of ESM-1 was reported in DA-resistant prolactinomas and null-cell adenomas. Moreover, the silencing of Endocan implied the downregulation of FGF2 and VEGFR2 in GH3 and MMQ cells; HUVECs reduced endothelial tube formations; and GH3 cells increased the sensitivity to Bevacizumab [248]. However, further studies are needed to identify the potential applicability of these preliminary results.

### 4.6. Genetic Aspects in Aggressive PitNETs

#### 4.6.1. Germline Mutations

Pituitary tumorigenesis may also be caused by germline mutations that are part of a syndromic presentation or in isolation. The first identified gene underlying familial isolated pituitary adenoma (FIPA) is AIP (Aryl hydrocarbon receptor interacting protein), which accounts for about 10–20% of FIPA kindreds [249]. It encodes a co-chaperone protein, which acts as a tumor suppressor, especially versus GH and PRL PitNETs. The prevalence of AIP mutations in patients with sporadic pituitary tumors is exceedingly rare, and it is higher among acromegalic patients and patients with less than 30 years of age at diagnosis [250]. Some data suggest that low AIP expression is a better marker of invasiveness in somatotropinomas than the Ki-67 labeling index and p53 [251]. Bogner et al. investigated miRNA dysregulation in AIP-mutated somatotropinomas, showing miR-34a overexpression in this subtype of tumor. Upregulation of miR-34a promotes cell proliferation by downregulating inhibitory G-protein alpha 2 protein expression, increasing cAMP concentration, and reducing PitNETs cell response to SSAs, including octreotides [252,253]. A low expression of SSTR2 was described in an acromegaly AIP-mutated patient [254]. During the last few years, further efforts have been made to fully understand the genetic and molecular pathways underlying AIP expression, trying to discover novel potential therapeutic targets. In vitro and in vivo experiments indicated that general transcription factor IIB (GTF2B) regulated AIP expression by binding a noncoding evolutionary conserved region (ncECR) within the 5′ untranslated region (UTR) of this gene [255].

#### 4.6.2. Somatic Mutations

Despite applying novel exome and whole genome sequencing techniques in the study of PitNETs, a few recurrent mutated genes have been reported [256,257]. Efforts in this respect will be paramount in developing novel therapeutic strategies and additional treatments for this kind of tumor. No apparent recurrent driver alterations have been identified for PitNETs, apart from GNAS and USP8 mutations in GH− and ACTH-PitNETs, respectively.

##### GNAS

GNAS is a complex imprinted gene characterized by several promoters. One of its products, the stimulatory α subunit of G-protein, is paramount in regulating the enzymatic activity of adenylyl cyclase. Tumor cell proliferation in PIT1 might be associated with GNAS copy number gain [258]. At the same time, mutations affecting GNAS codons 201 or 227 may lead to prolonged GTPase activity and increased cAMP levels, playing a role in GH secretion in acromegalic patients [259].

Increasing evidence suggests that GNAS-mutated tumors are more likely to be smaller, non-invasive, and better respond to SSAs [259,260,261,262], despite some authors showing no differences in tumor extension or response to SSAs between mutated and non-mutated tumors [263].

Moreover, dopamine receptor 2 seems to be overexpressed in GNAS-muted tumors, potentially predicting a higher response to dopamine agonists in this subtype of GH-PitNETs [256].

Both chemical inhibition and knockdown of GNAS have decreased the expression of cyclin proteins. Therefore, Zhang et al. suggested that inhibiting CDK-6 may represent a valuable therapeutic strategy for patients harboring a PIT-1 lineage tumor with GNAS mutations or copy number gain [258].

##### USP8

USP8, also known as ubiquitin-specific protease Y (UBPY), is a deubiquitinating enzyme displaying elevated activity toward EGFR and other receptor tyrosine kinases (RTKs) with similar signaling cascades [264,265]. USP8 gain-of-function mutations protected EGFR from lysosomal degradation, thereby increasing its expression. Interestingly, USP8 mutations have been only documented in sporadic Cushing’s disease. The most common somatic mutation involved exon 14, which encoded a putative binding domain [266]. Some evidence suggests that USP8 mutations contribute to ACTH hyperproduction. Patients with USP8-mutated PitNETs have been found to have similar levels of plasma ACTH to unmutated patients. Still, tumors were markedly smaller (<0.5 cm) and diffusely distributed within the sella turcica, making them difficult to detect by MRI scanning [265]. Ma et al. showed that USP8 knockdown attenuated ACTH secretion in primary USP8-mutated tumor cells [266].

USP8-mutated tumors are associated with an earlier onset and a higher risk of recurrence [267]. Retrospectively analyzing their cohort of patients, Albani et al. reported that the incidence of relapse over a 10-year follow-up in patients who obtained biochemical remission after surgery was higher in patients with tumors with the USP8 mutation [268]. In another cohort of pediatric patients with Cushing’s disease, recurrences have been documented in tumors with USP8 mutations [269]. Interestingly, in their paper, Wanichi et al. detected no recurrence within the first six months after surgery [270]. These data suggest that patients with USP8-mutated tumors may go into initial remission after surgery, even if they are more likely to show recurrence later in the clinical course. In addition, according to Hayashi et al., pituitary tumors harboring the USP8 mutation are prone to sphenoid invasion and an increased epithelial–mesenchymal-transition signature [271].

These data highlight the importance of USP8 as a potential therapeutic strategy for Cushing’s disease, especially for invasive and aggressive tumors. Moreover, anti-EGFR therapy (Gefitinib) seems more effective for patients carrying the USP8 mutation, attenuating ACTH secretion and cell proliferation. Recently, the USP8 mutation status has also been suggested as a predictor of response to pasireotide, a second-generation somatostatin analog with greater affinity for SSTR5 [272], whose expression has been found to be higher in USP8-mutated tumors [256].

### 4.7. Future Perspectives in PitNETs: The Role of Epigenetic

In the last decade, increasing evidence has suggested the importance of epigenetic modifications in influencing PitNET pathophysiology and clinical behavior. Indeed, most pituitary tumors are sporadic without any specific genetic driver mutations. Furthermore, a better understanding of epigenetic modifications in PitNETs is paramount to developing novel therapeutic strategies.

#### 4.7.1. DNA Methylation

The development of new DNA methylation detection and analysis techniques has provided novel information on the pathogenesis of pituitary tumors. In 2012, the first genome-wide analysis of the DNA methylome in PitNETs examined more than 14,000 genes [273]. Since its early applications, methylome profiling has appeared helpful in understanding PitNET pathophysiology. However, up to date, global DNA methylation profiling has failed to predict clinically significant differences among pituitary tumors [274].

DNA methylation is crucial in PitNETs pathophysiology, mainly driving the loss or reduced expression of tumor suppressor genes (TSGs) [275]. Many of those genes have been shown to harbor C-phosphate-G (CpG) island hypermethylation, determining their silencing. Ling et al. identified 34 CpGs on 17 genes, characterized as being hypomethylated in invasive NF-PitNETs compared to non-invasive ones. Interestingly, among differentially methylated genes FLT1 (Fms-related receptor tyrosine kinase 1) and SLIT3 (slit guidance ligand 3), most of them were associated with invasion and cell migration [276]. Cheng et al. suggested that DNA methylation and expression levels of FAM90A1 (family with sequence similarity 90 member A1) and ING2 (inhibitor of growth family member 2) may be able to predict tumor regrowth in patients harboring NF-PitNETs [277]. Kober et al. reported aberrant epigenetic deregulation of ITPKB (inositol-trisphosphate 3-kinase B) and CNKSR1 (connector enhancer of kinase suppressor of Ras 1), upregulated and downregulated, respectively, in invasive NF-PitNETs [274]. Wang et al. found LAMA2 (laminin subunit alpha 2) was downregulated in invasive non-functioning PitNETs, while its promoter was hypermethylated [278]. On the contrary, the authors suggested the overexpression and demethylation of LAMA2 to suppress tumor cell invasion via the PTEN-PI3K/AKT signaling pathway and MMP-9 inhibition [278].

#### 4.7.2. MicroRNA

MicroRNAs (miRs) are short, noncoding RNAs that contribute to post-transcriptional regulation of gene expression through several mechanisms. The role of miRs in PitNET pathophysiology is increasingly documented [279,280,281]. Nevertheless, contrasting evidence emerges from reviewing the literature, and further efforts should be made to clarify their role in future clinical practice. Many miRs have been reported to be dysregulated in PitNET patients. Writing on every miR is beyond the purposes of our paper. Herein, we focus on the main changes in miR expression found, which have been found in aggressive and invasive PitNETs compared with non-invasive ones and normal pituitary glands. Indeed, several miRs have been found to play a prominent role in driving tumor invasion in kindred tumors. Via whole genome-wide miR transcriptome profiling, Zhang et al. identified 31 upregulated miRs and 24 downregulated miRs to be somehow associated with PitNETs invasion [281]. Downregulation of miR-132 and miR-15a/16 with upregulation of SOX5 (SRY-box transcription factor 5) was documented in invasive tumors by Renjie et al. [282]. According to Du et al., MiR-145-5p expression (targeting TPT1, a translationally controlled tumor protein) was negatively associated with invasiveness in NF-PitNETs, promoting apoptosis through Bcl-xL downregulation and Bax upregulation [283]. Moreover, the Authors demonstrated that circOMA1 (hsa_circRNA_0002316) overexpression abrogated miR-145-5p and downregulated the TPT1 signaling pathway. Shen et al. found that MiR-543 was upregulated in invasive tumors [284]. Moreover, they also showed its overexpression in HP75 cells to increase cell proliferation, invasion, and migration and to reduce programmed cell death [284]. MiR-183, which acts on the cell cycle activator KIAA0101, was downregulated in aggressive PRL-PitNETs [285]. MiR 106b~25 cluster appeared to be overexpressed in invasive ACTH-PitNETs and Crooke cell adenomas [286]. These data have been confirmed by other research groups, which demonstrated that the upregulation of MiR-106b might influence the PI3K/AKT pathway, acting on pituitary tumor cell motility and invasion [72,287]. In their study, Palumbo et al. reported that MiR-26b (targeting PTEN) was upregulated in GH-PitNETs, while miR-128 (targeting BMI1) downregulation increased the invasiveness of pituitary tumor cells [288]. Moreover, MiR-338-3p expression was overexpressed in invasive GH-PitNETs, associated with PTTG1 upregulation [289].

Interestingly, a single miR may have different target genes. Duan et al. showed that the ectopic expression of miR-137, which is reduced in pituitary tumor tissues compared to normal controls, interfered with the proliferation and invasion of pituitary tumor cells via targeting AKT2 (AKT serine/threonine kinase 2) [290]. Moreover, Wei et al. reported miR-137 to target EGFR, thus inhibiting cell proliferation of GH3 cells and inducing apoptosis and G1-phase arrest. According to the authors, miR-137 mimic and AZD9291 (Osimertinib, an oral third-generation EGFR tyrosine kinase inhibitor)-mediated additive effects in GH3 cells [61]. Therefore, a combination of miR-137 and AZD9291 may represent a novel therapeutic strategy for PitNETs, especially when standard treatments fail [61].

In addition, according to Lei et al., miR-137 targeted MITF (melanocyte-inducing transcription factor) as well, playing an essential role in the Wnt-b-catenin signaling pathway, giving miR-137 a tumor-invasive suppressor function in prolactinomas [291]. It was demonstrated that, acting on the methylation status of the WIF1 (WNT inhibitory factor 1) promoter, MiRNA-137 also modulated the Wnt signaling pathway, as Song et al. showed in NF-PitNETs [292]. In that study, miRNA-374a-5p and miRNA-374b-5p were also overexpressed in the invasive group [292]. Interestingly, a single miR can play different roles in several tumor subtypes: miR-410-3p, for example, positively influencing proliferation and invasiveness in gonadotroph and corticotroph cells, increasing cyclin B1 levels, and activating MAPK, PTEN/AKT, and STAT3 signaling pathways [293]. Nevertheless, it appeared to not play a significant role in somatotrophs [293].

### 4.8. Molecular Target Therapy and Clinical Prospects

Molecular assessments, such as those described in the present work, could represent the key factor for the treatment of those subtypes of aggressive PitNETs that are not responsive to conventional therapies. In this view, the 2017 and 2022 WHO classifications of endocrine tumors tried to define and identify this subgroup of neoplasia [294,295]. In particular, the 2017 WHO classifications defined as “high-risk categories“ prolactinomas in males, poorly granulated somatotrophs, silent corticotrophs, Crooke cells, PIT1-positive silent plurihormonal PitNETs, and high Ki67% index PitNETs [294]. The recent update of the 2022 WHO PitNETs classification added the role of tumor cell lineages, distinguishing between immature and mature tumors of the PIT1 lineage and defining the inclusion features of the PIT1 lineage “family” [295]. Moreover, the analysis of antigens such as estrogen receptor alpha (ERα), members of zinc finger transcription regulatory auxiliary proteins (GATA3), and somatostatin receptor types 2 and 5 has recently been introduced as potential prognostic factors [295,296,297]. The 2022 WHO PitNETs classification integrated several pathology features to identify PitNETs with potential aggressive features. Sparsely granulated somatotropinomas typically occur in young acromegaly individuals and may be associated with genetic mutations, such as somatic GNAS or germline AIP mutations [295,298]. Densely granulated lactotroph tumors typically present as large PitNETs with very high prolactin levels and may show resistance to dopamine agonist therapy [295]. Immature PIT1-lineage tumors tend to be aggressive, regardless of whether their clinical presentation includes acromegaly, hyperthyroidism, or hyperprolactinemia, or they present as a clinically non-functioning tumor with lower rates of resectability and a propensity for tumor recurrence. Acidophil stem cell tumors (ASCTs) are typically resistant to dopamine agonist therapy and somatostatin analogues. Null cell adenoma, silent corticotroph adenoma, Crooke tumors, and sparsely granulated corticotroph adenomas are associated with increased aggressiveness. The 2022 WHO classification had a profound impact on therapeutic decision making. With a refined understanding of the molecular characteristics of PitNETs, clinicians can be more directed towards personalized therapy, increasing the chances of treatment response and improving patient outcomes [295,299].

## 5. Conclusions

Therefore, this comprehensive review synthesizes the molecular mechanisms in aggressive PitNETs that may open the door to related emerging target therapies. Although the described results are promising, further research must delve deeper into the molecular features of these challenging PitNETs to fully understand the potential of novel treatments.

## Figures and Tables

**Table 1:** List of target therapies currently available for PitNETs not responsive to conventional treatments, based on the molecular mechanism or specific pathway.

Molecular Mechanism/Pathway	Molecular Therapies
Immune-checkpoint inhibitors (ICIs)	Anti-CTLA-4: Ipilimumab Anti-PD-1/PD-L1: Nivolumab, Pembrolizumab
DNA repair mechanisms	Temozolomide
PI3K/Akt/mTOR and RAS/RAF/MEK/ERK pathways	mTOR Inhibitors: Rapamycin/Sirolimus, Everolimus/RAD001 PI3K Inhibitors: Buparlisib, Alpelisib PI3K-mTOR inhibitors: Dactolisib, Voxtalisib BRAF inhibitor: Vemurafenib
EGFR	anti-EGFR tyrosine kinase inhibitor: Gefitinib, Lapatinib
VEGF/VEGFR	VEGFA Inhibitor: Bevacizumab VEGFR Inhibitor: Sunitinib, Apatinib, Axitinib (also combined with Bromocriptine)
Estrogen Modulators	SERMs and SERDs with/without DA

## Data Availability

The authors confirm that the findings of this study are available within the article.

## References

[1] Melmed S. (2020). Pituitary-Tumor Endocrinopathies. N. Engl. J. Med..

[2] Saeger W., Lüdecke D.K., Buchfelder M., Fahlbusch R., Quabbe H.J., Petersenn S. (2007). Pathohistological classification of pituitary tumors: 10 years of experience with the German Pituitary Tumor Registry. Eur. J. Endocrinol..

[3] Trouillas J., Jaffrain-Rea M.L., Vasiljevic A., Raverot G., Roncaroli F., Villa C. (2020). How to Classify the Pituitary Neuroendocrine Tumors (PitNET)s in 2020. Cancers.

[4] Serioli S., Doglietto F., Fiorindi A., Biroli A., Mattavelli D., Buffoli B., Ferrari M., Cornali C., Rodella L., Maroldi R. (2019). Pituitary Adenomas and Invasiveness from Anatomo-Surgical, Radiological, and Histological Perspectives: A Systematic Literature Review. Cancers.

[5] Asa S.L., Casar-Borota O., Chanson P., Delgrange E., Earls P., Ezzat S., Grossman A., Ikeda H., Inoshita N., Karavitaki N. (2017). From pituitary adenoma to pituitary neuroendocrine tumor (PitNET): An International Pituitary Pathology Club proposal. Endocr. Relat. Cancer.

[6] Raverot G., Ilie M.D., Lasolle H., Amodru V., Trouillas J., Castinetti F., Brue T. (2021). Aggressive pituitary tumors and pituitary carcinomas. Nat. Rev. Endocrinol..

[7] Raverot G., Burman P., McCormack A., Heaney A., Petersenn S., Popovic V., Trouillas J., Dekkers O.M. (2018). European Society of Endocrinology. Clinical Practice Guidelines for the management of aggressive pituitary tumors and carcinomas. Eur. J. Endocrinol..

[8] Ilie M.D., Jouanneau E., Raverot G. (2020). Aggressive Pituitary Adenomas and Carcinomas. Endocrinol. Metab. Clin. N. Am..

[9] Ji Y., Vogel R.I., Lou E. (2016). Temozolomide treatment of pituitary carcinomas and atypical adenomas: Systematic review of case reports. Neurooncol. Pract..

[10] Lasolle H., Cortet C., Castinetti F., Cloix L., Caron P., Delemer B., Desailloud R., Jublanc C., Lebrun-Frenay C., Sadoul J.L. (2017). Temozolomide treatment can improve overall survival in aggressive pituitary tumors and pituitary carcinomas. Eur. J. Endocrinol..

[11] Chiloiro S., De Marinis L. (2023). The immune microenviroment in somatotropinomas: From biology to personalized and target therapy. Rev. Endocr. Metab. Disord..

[12] Ilie M.D., Vasiljevic A., Bertolino P., Raverot G. (2023). Biological and Therapeutic Implications of the Tumor Microenvironment in Pituitary Adenomas. Endocr. Rev..

[13] Yamamoto M., Nakao T., Ogawa W., Fukuoka H. (2021). Aggressive Cushing’s Disease: Molecular Pathology and Its Therapeutic Approach. Front. Endocrinol..

[14] Page M.J., McKenzie J.E., Bossuyt P.M., Boutron I., Hoffmann T.C., Mulrow C.D., Shamseer L., Tetzlaff J.M., Akl E.A., Brennan S.E. (2021). The PRISMA 2020 statement: An updated guideline for reporting systematic reviews. BMJ.

[15] DeLellis R.A., Lloyd R.V., Heitz P.U., Eng C. (2004). World Health Organization classification of tumors: Pathology and genetics of tumors of endocrine organs. Tumor of the Pituitary Gland.

[16] (2022). Critical Appraisal Skills Programme. CASP Checklist. https://casp-uk.net/casp-tools-checklists/.

[17] Lockwood C., Munn Z., Porritt K. (2015). Qualitative research synthesis: Methodological guidance for systematic reviewers utilizing meta-aggregation. Int. J. Evid. Based Healthc..

[18] Ilie M.D., Vasiljevic A., Raverot G., Bertolino P. (2019). The Microenvironment of Pituitary Tumors—Biological and Therapeutic Implications. Cancers.

[19] Zhang J., Gu C., Song Q., Zhu M., Xu Y., Xiao M., Zheng W. (2020). Identifying cancer-associated fibroblasts as emerging targets for hepatocellular carcinoma. Cell Biosci..

[20] Marques P., Korbonits M. (2023). Tumor microenvironment and pituitary tumor behaviour. J. Endocrinol. Investig..

[21] Principe M., Chanal M., Ilie M.D., Ziverec A., Vasiljevic A., Jouanneau E., Hennino A., Raverot G., Bertolino P. (2020). Immune landscape of pituitary tumors reveals association between macrophages and gonadotroph tumor invasion. J. Clin. Endocrinol. Metab..

[22] Zhou W., Zhang C., Zhang D., Peng J., Ma S., Wang X., Guan X., Li P., Li D., Jia G. (2020). Comprehensive analysis of the immunological landscape of pituitary adenomas: Implications of immunotherapy for pituitary adenomas. J. Neurooncol..

[23] Lyu L., Jiang Y., Ma W., Li H., Liu X., Li L., Shen A., Yu Y., Jiang S., Li H. (2023). Single-cell sequencing of PIT1- positive pituitary adenoma highlights the pro-tumor microenvironment mediated by IFN-gamma-induced tumor-associated fbroblasts remodelling. Br. J. Cancer.

[24] Heshmati H.M., Kujas M., Casanova S., Wollan P.C., Racadot J., VAN Effenterre R., Derome P.J., Turpin G. (1998). Prevalence of lymphocytic infiltrate in 1400 pituitary adenomas. Endocr. J..

[25] Marques P., Barry S., Carlsen E., Collier D., Ronaldson A., Awad S., Dorward N., Grieve J., Mendoza N., Muquit S. (2019). Chemokines modulate the tumor microenvironment in pituitary neuroendocrine tumors. Acta Neuropathol. Commun..

[26] Huang X., Xu J., Wu Y., Sheng L., Li Y., Zha B., Sun T., Yang J., Zang S., Liu J. (2021). Alterations in CD8+ Tregs, CD56+ Natural Killer Cells and IL-10 Are Associated with Invasiveness of Nonfunctioning Pituitary Adenomas (NFPAs). Pathol. Oncol. Res..

[27] Wang P.-F., Wang T.-J., Yang Y.-K., Yao K., Li Z., Li Y.M., Yan C.X. (2018). The expression profile of PD-L1 and CD8(+) lymphocyte in pituitary adenomas indicating for immunotherapy. J. Neuro Oncol..

[28] Mei Y., Bi W.L., Agolia J., Hu C., Larsen A.M.G., Meredith D.M., Al Abdulmohsen S., Bale T., Dunn G.P., Abedalthagafi M. (2021). Immune profiling of pituitary tumors reveals variations in immune infiltration and checkpoint molecule expression. Pituitary.

[29] Moldovan I.M., Şuşman S., Pîrlog R., Jianu E.M., Leucuţa D.C., Melincovici C.S., Crişan D., Florian I.Ş. (2017). Molecular markers in the diagnosis of invasive pituitary adenomas—An immunohistochemistry study. Rom. J. Morphol. Embryol..

[30] Qu X., Yang W., Jiang M., Han T., Han L., Qu Y., Wang G., Shi D., Xu G. (2010). CD147 expression in pituitary adenomas and its significance for clinical outcome. Hum. Pathol..

[31] Lv L., Zhang S., Hu Y., Zhou P., Gao L., Wang M., Sun Z., Chen C., Yin S., Wang X. (2018). Invasive pituitary adenomaderived tumor-associated fibroblasts promote tumor progression both in vitro and in vivo. Exp. Clin. Endocrinol. Diabetes.

[32] Azorín E., Solano-Agama C., Mendoza-Garrido M.E. (2012). The invasion mode of GH(3) cells is conditioned by collagen subtype, and its efficiency depends on cell-cell adhesion. Arch. Biochem. Biophys..

[33] Marques P., Barry S., Carlsen E., Collier D., Ronaldson A., Awad S., Dorward N., Grieve J., Mendoza N., Muquit S. (2019). Pituitary tumor fibroblast derived cytokines influence tumor aggressiveness. Endocr. Relat. Cancer.

[34] Zhang D., Hugo W., Bergsneider M., Wang M.B., Kim W., Vinters H.V., Heaney A.P. (2022). Single-cell RNA sequencing in silent corticotroph tumors confirms impaired POMC processing and provides new insights into their invasive behavior. Eur. J. Endocrinol..

[35] Devnath S., Inoue K. (2008). An insight to pituitary folliculo-stellate cells. J. Neuroendocr..

[36] Ilie M.D., Vasiljevic A., Chanal M., Gadot N., Chinezu L., Jouanneau E., Hennino A., Raverot G., Bertolino P. (2022). Intratumoral spatial distribution of S100B + folliculostellate cells is associated with proliferation and expression of FSH and ERalpha in gonadotroph tumors. Acta Neuropathol. Commun..

[37] Voit D., Saeger W., Lüdecke D.K. (1999). Folliculo-stellate cells in pituitary adenomas of patients with acromegaly. Pathol. Res. Pract..

[38] Zhang A., Xu Y., Xu H., Ren J., Meng T., Ni Y., Zhu Q., Zhang W.B., Pan Y.B., Jin J. (2021). Lactate-induced M2 polarization of tumor-associated macrophages promotes the invasion of pituitary adenoma by secreting CCL17. Theranostics.

[39] Kim Y.H., Kim J.H. (2019). Transcriptome Analysis Identifies an Attenuated Local Immune Response in Invasive Nonfunctioning Pituitary Adenomas. Endocrinol. Metab..

[40] Wang Q., Lei Z., Wang Z., Jiang Q., Zhang Z., Liu X., Xing B., Li S., Guo X., Liu Y. (2023). PKCθ Regulates Pituitary Adenoma Bone Invasion by Activating Osteoclast in NF-κB/IL-1β-Dependent Manner. Cancers.

[41] Voellger B., Zhang Z., Benzel J., Wang J., Lei T., Nimsky C., Bartsch J.W. (2021). Targeting Aggressive Pituitary Adenomas at the Molecular Level—A Review. J. Clin. Med..

[42] Duhamel C., Ilie M.D., Salle H., Nassouri A.S., Gaillard S., Deluche E., Assaker R., Mortier L., Cortet C., Raverot G. (2020). Immunotherapy in Corticotroph and Lactotroph Aggressive Tumors and Carcinomas: Two Case Reports and a Review of the Literature. J. Pers. Med..

[43] Majd N., Waguespack S.G., Janku F., Fu S., Penas-Prado M., Xu M., Alshawa A., Kamiya-Matsuoka C., Raza S.M., McCutcheon I.E. (2020). Efficacy of pembrolizumab in patients with pituitary carcinoma: Report of four cases from a phase II study. J. Immunother. Cancer.

[44] Lin A.L., Tabar V., Young R.J., Cohen M., Cuaron J., Yang T.J., Rosenblum M., Rudneva V.A., Geer E.B., Bodei L. (2021). Synergism of Checkpoint Inhibitors and Peptide Receptor Radionuclide Therapy in the Treatment of Pituitary Carcinoma. J. Endocr. Soc..

[45] Chiloiro S., Giampietro A., Gessi M., Lauretti L., Mattogno P.P., Cerroni L., Carlino A., De Alessandris Q.G., Olivi A., Rindi G. (2023). CD68+ and CD8+ immune cells are associated with the growth pattern of somatotroph tumors and response to first generation somatostatin analogs. J. Neuroendocr..

[46] Yang Z., Tian X., Yao K., Yang Y., Zhang L., Liu N., Yan C., Qi X., Han S. (2023). Targeting the Tumor Immune Microenvironment Could Become a Potential Therapeutic Modality for Aggressive Pituitary Adenoma. Brain Sci..

[47] Matsuzaki H., Komohara Y., Yano H., Fujiwara Y., Kai K., Yamada R., Yoshii D., Uekawa K., Shinojima N., Mikami Y. (2022). Macrophage colony-stimulating factor potentially induces recruitment and maturation of macrophages in recurrent pituitary neuroendocrine tumors. Microbiol. Immunol..

[48] Ilie M.D., Villa C., Cuny T., Cortet C., Assie G., Baussart B., Cancel M., Chanson P., Decoudier B., Deluche E. (2022). Real-life efficacy and predictors of response to immunotherapy in pituitary tumors: A cohort study. Eur. J. Endocrinol..

[49] Xi Z., Jones P.S., Mikamoto M., Jiang X., Faje A.T., Nie C., Labelle K.E., Zhou Y., Miller K.K., Soberman R.J. (2021). The upregulation of molecules related to tumor immune escape in human pituitary adenomas. Front. Endocrinol..

[50] Thiele J.O., Lohrer P., Schaaf L., Feirer M., Stummer W., Losa M., Lange M., Tichomirowa M., Arzt E., Stalla G.K. (2003). Functional in vitro studies on the role and regulation of interleukin-6 in human somatotroph pituitary adenomas. Eur. J. Endocrinol..

[51] Zatelli M.C., Piccin D., Vignali C., Tagliati F., Ambrosio M.R., Bondanelli M., Cimino V., Bianchi A., Schmid H.A., Scanarini M. (2007). Pasireotide, a multiple somatostatin receptor subtypes ligand, reduces cell viability in non-functioning pituitary adenomas by inhibiting vascular endothelial growth factor secretion. Endocr. Relat. Cancer.

[52] Beck G.C.H., Brinkkoetter P., Hanusch C., Schulte J., van Ackern K., van der Woude F.J., Yard B.A. (2004). Clinical review: Immunomodulatory effects of dopamine in general inflammation. Crit. Care.

[53] Casanueva F.F., Molitch M.E., Schlechte J.A., Abs R., Bonert V., Bronstein M.D., Brue T., Cappabianca P., Colao A., Fahlbusch R. (2006). Guidelines of the Pituitary Society for the diagnosis and management of prolactinomas. Clin. Endocrinol..

[54] Recouvreux M.V., Camilletti M.A., Rifkin D.B., Díaz-Torga G. (2016). The pituitary TGFbeta1 system as a novel target for the treatment of resistant prolactinomas. J. Endocrinol..

[55] Chauvet N., Romanò N., Lafont C., Guillou A., Galibert E., Bonnefont X., Le Tissier P., Fedele M., Fusco A., Mollard P. (2017). Complementary actions of dopamine D2 receptor agonist and anti-vegf therapy on tumoral vessel normalization in a transgenic mouse model. Int. J. Cancer.

[56] Zhao S., Li B., Chen Y., Li C., Zhang Y. (2023). Analysis of the Prognostic and Immunological Role of HSPB1 in Pituitary Adenoma: A Potential Target for Therapy. Medicina.

[57] Saxton R.A., Sabatini D.M. (2017). mTOR Signaling in Growth, Metabolism, and Disease. Cell.

[58] McCubrey J.A., Steelman L.S., Chappell W.H., Abrams S.L., Wong E.W., Chang F., Lehmann B., Terrian D.M., Milella M., Tafuri A. (2007). Roles of the Raf/MEK/ERK pathway in cell growth, malignant transformation and drug resistance. Biochim. Biophys. Acta.

[59] Guo Y.J., Pan W.W., Liu S.B., Shen Z.F., Xu Y., Hu L.L. (2020). ERK/MAPK signalling pathway and tumorigenesis. Exp. Ther. Med..

[60] Vlotides G., Siegel E., Donangelo I., Gutman S., Ren S.G., Melmed S. (2008). Rat Prolactinoma cell growth regulation by Epidermal Growth Factor receptor ligands. Cancer Res..

[61] Wei D., Yu Z., Cheng Y., Jiawei H., Jian G., Hua G., Guilan D. (2021). Dysregulated miR-137 and its target EGFR contribute to the progression of pituitary adenomas. Mol. Cell Endocrinol..

[62] Wei D., Yu Z., Cheng Y., Jiawei H., Jian G., Hua G., Guilan D. (2018). ADAM12 induces EMT and promotes cell migration, invasion and proliferation in pituitary adenomas via EGFR/ERK signaling pathway. Biomed. Pharmacother..

[63] Rose A., Froment P., Perrot V., Quon M.J., LeRoith D., Dupont J. (2004). The Luteinizing Hormone-releasing Hormone Inhibits the Anti-apoptotic Activity of Insulin-like Growth Factor-1 in Pituitary T3 Cells by Protein Kinase C-mediated Negative Regulation of Akt. J. Biol. Chem..

[64] Fernández M., Sánchez-Franco F., Palacios N., Sánchez I., Fernández C., Cacicedo L. (2004). IGF-I inhibits apoptosis through the activation of the phosphatidylinositol 3-kinase/Akt pathway in pituitary cells. J. Mol. Endocrinol..

[65] Kowarik M., Onofri C., Colaco T., Stalla G.K., Renner U. (2010). Platelet-derived Growth Factor (PDGF) and PDGF Receptor Expression and Function in Folliculostellate Pituitary Cells. Exp. Clin. Endocrinol. Diabetes.

[66] Murat C.B., Braga P.B., Fortes M.A., Bronstein M.D., Corrêa-Giannella M.L., Giorgi R.R. (2012). Mutation and genomic amplification of the PIK3CA proto-oncogene in pituitary adenomas. Braz. J. Med. Biol. Res..

[67] Lin Y., Jiang X., Shen Y., Li M., Ma H., Xing M., Lu Y. (2009). Frequent mutations and amplifications of the PIK3CA gene in pituitary tumors. Endocr. Relat. Cancer.

[68] Musat M., Korbonits M., Kola B., Borboli N., Hanson M.R., Nanzer A.M., Grigson J., Jordan S., Morris D.G., Gueorguiev M. (2005). Enhanced protein kinase B/Akt signalling in pituitary tumors. Endocr. Relat. Cancer.

[69] Jia W., Sanders A.J., Jia G., Liu X., Lu R., Jiang W.G. (2013). Expression of the mTOR pathway regulators in human pituitary adenomas indicates the clinical course. Anticancer Res..

[70] Xu Q., Yu Z.X., Xie Y.L., Bai L., Liang S.R., Ji Q.H., Zhou J. (2023). MicroRNA-137 inhibits pituitary prolactinoma proliferation by targeting AKT2. J. Endocrinol. Investig..

[71] Noh T.W., Jeong H.J., Lee M.K., Kim T.S., Kim S.H., Lee E.J. (2009). Predicting Recurrence of Nonfunctioning Pituitary Adenomas. J. Clin. Endocrinol. Metab..

[72] Zhou K., Zhang T., Fan Y., Serick Du G., Wu P., Geng D. (2016). MicroRNA-106b promotes pituitary tumor cell proliferation and invasion through PI3K/AKT signaling pathway by targeting PTEN. Tumor Biol..

[73] Zhang D. (2019). Effect of Everolimus in Treatment of Aggressive Prolactin-Secreting Pituitary Adenomas. J. Clin. Endocrinol. Metab..

[74] Lu C., Willingham M.C., Furuya F., Cheng S.Y. (2008). Activation of Phosphatidylinositol 3-Kinase Signaling Promotes Aberrant Pituitary Growth in a Mouse Model of Thyroid-Stimulating Hormone-Secreting Pituitary Tumors. Endocrinology.

[75] Gorshtein A., Rubinfeld H., Kendler E., Theodoropoulou M., Cerovac V., Stalla G.K., Cohen Z.R., Hadani M., Shimon I. (2009). Mammalian target of rapamycin inhibitors rapamycin and RAD001 (everolimus) induce anti-proliferative effects in GH-secreting pituitary tumor cells in vitro. Endocr. Relat. Cancer.

[76] Sajjad E.A., Zieliński G., Maksymowicz M., Hutnik Ł., Bednarczuk T., Włodarski P. (2013). mTOR is Frequently Active in GH-Secreting Pituitary Adenomas without Influencing their Morpho-pathological Features. Endocr. Pathol..

[77] Dworakowska D., Wlodek E., Leontiou C.A., Igreja S., Cakir M., Teng M., Prodromou N., Góth M.I., Grozinsky-Glasberg S., Gueorguiev M. (2009). Activation of RAF/MEK/ERK and PI3K/AKT/mTOR pathways in pituitary adenomas and their effects on downstream effectors. Endocr. Relat. Cancer.

[78] Yao H., Tang H., Zhang Y., Zhang Q.F., Liu X.Y., Liu Y.T., Gu W.T., Zheng Y.Z., Shang H.B., Wang Y. (2019). DEPTOR inhibits cell proliferation and confers sensitivity to dopamine agonist in pituitary adenoma. Cancer Lett..

[79] Jian F., Chen Y., Ning G., Fu W., Tang H., Chen X., Zhao Y., Zheng L., Pan S., Wang W. (2016). Cold inducible RNA binding protein upregulation in pituitary corticotroph adenoma induces corticotroph cell proliferation via Erk signaling pathway. Oncotarget.

[80] Cheng S.Q., Fan H.Y., Xu X., Gao W.W., Lv S.G., Ye M.H., Wu M.J., Shen X.L., Cheng Z.J., Zhu X.G. (2016). Over-expression of LRIG1 Suppresses Biological Function of Pituitary Adenoma via Attenuation of PI3K/AKT and Ras/Raf/ERK Pathways In Vivo and In Vitro. J. Huazhong Univ. Sci. Technol. Med. Sci..

[81] Liu J., Wang J., Tian W., Xu Y., Li R., Zhao K., You C., Zhu Y., Bartsch J.W., Niu H. (2022). PDCD10 promotes the aggressive behaviors of pituitary adenomas by up-regulating CXCR2 and activating downstream AKT/ERK signaling. Aging.

[82] Romano D., Pertuit M., Rasolonjanahary R., Barnier J.V., Magalon K., Enjalbert A., Gerard C. (2006). Regulation of the RAP1/RAF-1/Extracellularly Regulated Kinase-1/2 Cascade and Prolactin Release by the Phosphoinositide 3-Kinase/AKT Pathway in Pituitary Cells. Endocrinology.

[83] Ewing I., Pedder-Smith S., Franchi G., Ruscica M., Emery M., Vax V., Garcia E., Czirják S., Hanzély Z., Kola B. (2007). A mutation and expression analysis of the oncogene BRAF in pituitary adenomas. Clin. Endocrinol..

[84] Chen J. (2018). Identification of recurrent USP48 and BRAF mutations in Cushing’s disease. Nat. Commun..

[85] Pei L., Melmed S., Scheithauer B., Kovacs K., Prager D. (1994). H-ras mutations in human pituitary carcinoma metastases. J. Clin. Endocrinol. Metab..

[86] Sukumari-Ramesh S., Singh N., Dhandapani K.M., Vender J.R. (2011). mTOR inhibition reduces cellular proliferation and sensitizes pituitary adenoma cells to ionizing radiation. Surg. Neurol. Int..

[87] Pivonello C., Patalano R., Solari D., Auriemma R.S., Frio F., Vitulli F., Grasso L.F.S., Di Cera M., De Martino M.C., Cavallo L.M. (2018). Effect of combined treatment with a pan-PI3K inhibitor or an isoform-specific PI3K inhibitor and everolimus on cell proliferation in GH-secreting pituitary tumor in an experimental setting. Endocrine.

[88] Zatelli M.C., Minoia M., Filieri C., Tagliati F., Buratto M., Ambrosio M.R., Lapparelli M., Scanarini M., Degli Uberti E.C. (2010). Effect of Everolimus on Cell Viability in Nonfunctioning Pituitary Adenomas. J. Clin. Endocrinol. Metab..

[89] Cerovac V., Monteserin-Garcia J., Rubinfeld H., Buchfelder M., Losa M., Florio T., Paez-Pereda M., Stalla G.K., Theodoropoulou M. (2010). The Somatostatin Analogue Octreotide Confers Sensitivity to Rapamycin Treatment on Pituitary Tumor Cells. Cancer Res..

[90] Theodoropoulou M., Zhang J., Laupheimer S., Paez-Pereda M., Erneux C., Florio T., Pagotto U., Stalla G.K. (2006). Octreotide, a Somatostatin Analogue, Mediates Its Antiproliferative Action in Pituitary Tumor Cells by Altering Phosphatidylinositol 3-Kinase Signaling and Inducing Zac1 Expression. Cancer Res..

[91] Chanal M., Chevallier P., Raverot V., Fonteneau G., Lucia K., Garcia J.L.M., Rachwan A., Jouanneau E., Trouillas J., Honnorat J. (2016). Differential Effects of PI3K and Dual PI3K/mTOR Inhibition in Rat Prolactin-Secreting Pituitary Tumors. Mol. Cancer Ther..

[92] Wang J., Zhang J., Ma D., Li X. (2020). The Potential Role of CERS1 in Autophagy Through PI3K/AKT Signaling Pathway in Hypophysoma. Technol. Cancer Res. Treat..

[93] Lee M., Theodoropoulou M., Graw J., Roncaroli F., Zatelli M.C., Pellegata N.S. (2011). Levels of p27 Sensitize to Dual PI3K/mTOR Inhibition. Mol. Cancer Ther..

[94] Lee M., Wiedemann T., Gross C., Leinhäuser I., Roncaroli F., Braren R., Pellegata N.S. (2015). Targeting PI3K/mTOR Signaling Displays Potent Antitumor Efficacy against Nonfunctioning Pituitary Adenomas. Clin. Cancer Res..

[95] Dai C., Zhang B., Liu X., Ma S., Yang Y., Yao Y., Feng M., Bao X., Li G., Wang J. (2013). Inhibition of PI3K/AKT/mTOR Pathway Enhances Temozolomide-Induced Cytotoxicity in Pituitary Adenoma Cell Lines in Vitro and Xenografted Pituitary Adenoma in Female Nude Mice. Endocrinology.

[96] Zeng J., See A.P., Aziz K., Thiyagarajan S., Salih T., Gajula R.P., Armour M., Phallen J., Terezakis S., Kleinberg L. (2011). Nelfinavir induces radiation sensitization in pituitary adenoma cells. Cancer Biol. Ther..

[97] Reubi J.C., Waser B., Schaer J.C., Laissue J.A. (2001). Somatostatin receptor sst1-sst5 expression in normal and neoplastic human tissues using receptor autoradiography with subtype-selective ligands. Eur. J. Nucl. Med..

[98] Behling F., Honegger J., Skardelly M., Gepfner-Tuma I., Tabatabai G., Tatagiba M., Schittenhelm J. (2018). High Expression of Somatostatin Receptors 2A, 3, and 5 in Corticotroph Pituitary Adenoma. Int. J. Endocrinol..

[99] Magagna-Poveda A., Leske H., Schmid C., Bernays R., Rushing E.J. (2013). Expression of somatostatin receptors, angiogenesis and proliferation markers in pituitary adenomas: An immunohistochemical study with diagnostic and therapeutic implications. Swiss Med. Wkly..

[100] Ramírez C., Cheng S., Vargas G., Asa S.L., Ezzat S., González B., Cabrera L., Guinto G., Mercado M. (2012). Expression of Ki-67, PTTG1, FGFR4, and SSTR 2, 3, and 5 in nonfunctioning pituitary adenomas: A high throughput TMA, immunohistochemical study. J. Clin. Endocrinol. Metab..

[101] Brzana J., Yedinak C.G., Gultekin S.H., Delashaw J.B., Fleseriu M. (2013). Growth hormone granulation pattern and somatostatin receptor subtype 2A correlate with postoperative somatostatin receptor ligand response in acromegaly: A large single center experience. Pituitary.

[102] Venegas-Moreno E., Vazquez-Borrego M.C., Dios E., Gros-Herguido N., Flores-Martinez A., Rivero-Cortés E., Madrazo-Atutxa A., Japón M.A., Luque R.M., Castaño J.P. (2018). Association between dopamine and somatostatin receptor expression and pharmacological response to somatostatin analogues in acromegaly. J. Cell Mol. Med..

[103] Peverelli E., Giardino E., Treppiedi D., Catalano R., Mangili F., Locatelli M., Lania A.G., Arosio M., Spada A., Mantovani G. (2018). A novel pathway activated by somatostatin receptor type 2 (SST2): Inhibition of pituitary tumor cell migration and invasion through cytoskeleton protein recruitment. Int. J. Cancer.

[104] Peverelli E., Giardino E., Treppiedi D., Locatelli M., Vaira V., Ferrero S., Bosari S., Lania A.G., Spada A., Mantovani G. (2016). Dopamine receptor type 2 (DRD2) inhibits migration and invasion of human tumorous pituitary cells through ROCK-mediated cofilin inactivation. Cancer Lett..

[105] Boscaro M., Ludlam W.H., Atkinson B., Glusman J.E., Petersenn S., Reincke M., Snyder P., Tabarin A., Biller B.M., Findling J. (2009). Treatment of pituitary-dependent Cushing’s disease with the multireceptor ligand somatostatin analog pasireotide (SOM230): A multicenter, phase II trial. J. Clin. Endocrinol. Metab..

[106] Moreno-Moreno P., Ibáñez-Costa A., Venegas-Moreno E., Fuentes-Fayos A.C., Alhambra-Expósito M.R., Fajardo-Montañana C., García-Martínez A., Dios E., Vázquez-Borrego M.C., Remón-Ruiz P. (2022). Integrative Clinical, Radiological, and Molecular Analysis for Predicting Remission and Recurrence of Cushing Disease. J. Clin. Endocrinol. Metab..

[107] Luque R.M., Ibáñez-Costa A., Neto L.V., Taboada G.F., Hormaechea-Agulla D., Kasuki L., Venegas-Moreno E., Moreno-Carazo A., Gálvez M.Á., Soto-Moreno A. (2015). Truncated somatostatin receptor variant sst5TMD4 confers aggressive features (proliferation, invasion and reduced octreotide response) to somatotropinomas. Cancer Lett..

[108] Ibáñez-Costa A., López-Sánchez L.M., Gahete M.D., Rivero-Cortés E., Vázquez-Borrego M.C., Gálvez M.A., de la Riva A., Venegas-Moreno E., Jiménez-Reina L., Moreno-Carazo A. (2017). BIM-23A760 influences key functional endpoints in pituitary adenomas and normal pituitaries: Molecular mechanisms underlying the differential response in adenomas. Sci. Rep..

[109] De Bruin C., Pereira A.M., Feelders R.A., Romijn J.A., Roelfsema F., Sprij-Mooij D.M., van Aken M.O., van der Lelij A.J., de Herder W.W., Lamberts S.W. (2009). Coexpression of dopamine and somatostatin receptor subtypes in corticotroph adenomas. J. Clin. Endocrinol. Metab..

[110] Lee M., Lupp A., Mendoza N., Martin N., Beschorner R., Honegger J., Schlegel J., Shively T., Pulz E., Schulz S. (2015). SSTR3 is a putative target for the medical treatment of gonadotroph adenomas of the pituitary. Endocr. Relat. Cancer.

[111] Hofland J., Brabander T., Verburg F.A., Feelders R.A., de Herder W.W. (2022). Peptide Receptor Radionuclide Therapy. J. Clin. Endocrinol. Metab..

[112] Gonzalez P., Debnath S., Chen Y.A., Hernandez E., Jha P., Dakanali M., Hsieh J.T., Sun X. (2023). A Theranostic Small-Molecule Prodrug Conjugate for Neuroendocrine Prostate Cancer. Pharmaceutics.

[113] Gupta S.K., Singla S., Damle N.A., Agarwal K., Bal C. (2012). Diagnosis of Men-I Syndrome on (68)Ga-DOTANOC PET-CT and Role of Peptide Receptor Radionuclide Therapy with (177)Lu-DOTATATE. Int. J. Endocrinol. Metab..

[114] Baldari S., Ferraù F., Alafaci C., Herberg A., Granata F., Militano V., Salpietro F.M., Trimarchi F., Cannavò S. (2012). First demonstration of the effectiveness of peptide receptor radionuclide therapy (PRRT) with 111In-DTPA-octreotide in a giant PRL-secreting pituitary adenoma resistant to conventional treatment. Pituitary.

[115] Giuffrida G., Ferraù F., Laudicella R., Cotta O.R., Messina E., Granata F., Angileri F.F., Vento A., Alibrandi A., Baldari S. (2019). Peptide receptor radionuclide therapy for aggressive pituitary tumors: A monocentric experience. Endocr. Connect..

[116] Priola S.M., Esposito F., Cannavò S., Conti A., Abbritti R.V., Barresi V., Baldari S., Ferraù F., Germanò A., Tomasello F. (2017). Aggressive Pituitary Adenomas: The Dark Side of the Moon. World Neurosurg..

[117] Kovács G.L., Góth M., Rotondo F., Scheithauer B.W., Carlsen E., Saadia A., Hubina E., Kovács L., Szabolcs I., Nagy P. (2013). ACTH-secreting Crooke cell carcinoma of the pituitary. Eur. J. Clin. Investig..

[118] Komor J., Reubi J.C., Christ E.R. (2014). Peptide receptor radionuclide therapy in a patient with disabling non-functioning pituitary adenoma. Pituitary.

[119] Maclean J., Aldridge M., Bomanji J., Short S., Fersht N. (2014). Peptide receptor radionuclide therapy for aggressive atypical pituitary adenoma/carcinoma: Variable clinical response in preliminary evaluation. Pituitary.

[120] Bengtsson D., Schrøder H.D., Andersen M., Maiter D., Berinder K., Rasmussen U.F., Rasmussen Å.K., Johannsson G., Hoybye C., van der Lely A.J. (2015). Long-term outcome and MGMT as a predictive marker in 24 patients with atypical pituitary adenomas and pituitary carcinomas given treatment with temozolomide. J. Clin. Endocrinol. Metab..

[121] Burman P., Trouillas J., Losa M., McCormack A., Petersenn S., Popovic V., Theodoropoulou M., Raverot G., Dekkers O.M., ESE Survey Collaborators (2022). Aggressive pituitary tumors and carcinomas, characteristics and management of 171 patients. Eur. J. Endocrinol..

[122] Novruzov F., Aliyev A., Wan M.Y.S., Syed R., Mehdi E., Aliyeva I., Giammarile F., Bomanji J.B., Kayani I. (2021). The value of [68Ga]Ga-DOTA-TATE PET/CT in diagnosis and management of suspected pituitary tumors. Eur. J. Hybrid Imaging.

[123] Waligórska-Stachura J., Gut P., Sawicka-Gutaj N., Liebert W., Gryczyńska M., Baszko-Błaszyk D., Blanco-Gangoo A.R., Ruchała M. (2016). Growth hormone-secreting macroadenoma of the pituitary gland successfully treated with the radiolabeled somatostatin analog (90)Y-DOTATATE: Case report. J. Neurosurg..

[124] Assadi M., Nemati R., Shooli H., Rekabpour S.J., Nabipour I., Jafari E., Gholamrezanezhad A., Amini A., Ahmadzadehfar H. (2020). An aggressive functioning pituitary adenoma treated with peptide receptor radionuclide therapy. Eur. J. Nucl. Med. Mol. Imaging.

[125] Guan B., Zhou N., Wu C.-Y., Li S., Chen Y.-A., Debnath S., Hofstad M., Ma S., Raj G.V., He D. (2021). Validation of SV2A-Targeted PET Imaging for Noninvasive Assessment of Neuroendocrine Differentiation in Prostate Cancer. Int. J. Mol. Sci..

[126] Shi Y., Massagué J. (2003). Mechanisms of TGF-beta signaling from cell membrane to the nucleus. Cell.

[127] Massagué J. (2008). TGFbeta in Cancer. Cell.

[128] Jiang M., Mou C.Z., Han T., Wang M., Yang W. (2012). Thrombospondin-1 and transforming growth factor-β1 levels in prolactinoma and their clinical significance. J. Int. Med. Res..

[129] Elenkova A., Atanassova I., Kirilov G., Vasilev V., Kalinov K., Zacharieva S. (2013). Transforming growth factor β1 is not a reliable biomarker for valvular fibrosis but could be a potential serum marker for invasiveness of prolactinomas (pilot study). Eur. J. Endocrinol..

[130] Dallago C.M., Barbosa-Coutinho L.M., Ferreira N.P., Meurer R., Pereira-Lima J.F., Oliveira M.d.C. (2010). Determination of cell proliferation using Mcm2 antigen and evaluation of apoptosis and TGF-beta1 expression in GH-secreting or clinically nonfunctioning pituitary adenomas. Endocr. Pathol..

[131] Zhu H., Yao X., Wu L., Li C., Bai J., Gao H., Ji H., Zhang Y. (2018). Association of TGF-β1 and WIF1 Expression with 36 Paired Primary/Recurrent Nonfunctioning Pituitary Adenomas: A High-Throughput Tissue Microarrays Immunohistochemical Study. World Neurosurg..

[132] Duan J., Hu C., Zhang Q., Zhu J. (2022). Exploration of the Effects of TGF-β Pathway-Based Pituitary Tumor of Rats on GH3 Cell Line after Intervention with Different Concentrations of TGZ. Contrast Media Mol. Imaging.

[133] Gu Y.H., Feng Y.G. (2018). Down-regulation of TGF-β RII expression is correlated with tumor growth and invasion in non-functioning pituitary adenomas. J. Clin. Neurosci..

[134] Petiti J.P., Sosa L.D.V., Picech F., Crespo G.D.M., Rojas J.Z.A., Pérez P.A., Guido C.B., Leimgruber C., Sabatino M.E., García P. (2018). Trastuzumab inhibits pituitary tumor cell growth modulating the TGFB/SMAD2/3 pathway. Endocr. Relat. Cancer.

[135] Liu C., Li Z., Wu D., Li C., Zhang Y. (2016). Smad3 and phospho-Smad3 are potential markers of invasive nonfunctioning pituitary adenomas. Onco Targets Ther..

[136] Eswarakumar V.P., Lax I., Schlessinger J. (2005). Cellular signaling by fibroblast growth factor receptors. Cytokine Growth Factor. Rev..

[137] Giraldi F.P., Moro M., Cavagnini F., Study Group on the Hypothalamo-Pituitary-Adrenal Axis of the Italian Society of Endocrinology (2003). Gender-related differences in the presentation and course of Cushing’s disease. J. Clin. Endocrinol. Metab..

[138] Qian Z.R., Sano T., Asa S.L., Yamada S., Horiguchi H., Tashiro T., Li C.C., Hirokawa M., Kovacs K., Ezzat S. (2004). Cytoplasmic expression of fibroblast growth factor receptor-4 in human pituitary adenomas: Relation to tumor type, size, proliferation, and invasiveness. J. Clin. Endocrinol. Metab..

[139] Brito L.P., Lerário A.M., Bronstein M.D., Soares I.C., Mendonca B.B., Fragoso M.C. (2010). Influence of the fibroblast growth factor receptor 4 expression and the G388R functional polymorphism on Cushing’s disease outcome. J. Clin. Endocrinol. Metab..

[140] Morita K., Takano K., Yasufuku-Takano J., Yamada S., Teramoto A., Takei M., Osamura R.Y., Sano T., Fujita T. (2008). Expression of pituitary tumor-derived, N-terminally truncated isoform of fibroblast growth factor receptor 4 (ptd-FGFR4) correlates with tumor invasiveness but not with G-protein alpha subunit (gsp) mutation in human GH-secreting pituitary adenomas. Clin. Endocrinol..

[141] Ezzat S., Zheng L., Winer D., Asa S.L. (2006). Targeting N-cadherin through fibroblast growth factor receptor-4: Distinct pathogenetic and therapeutic implications. Mol. Endocrinol..

[142] Nawaz F.Z., Kipreos E.T. (2022). Emerging roles for folate receptor FOLR1 in signaling and cancer. Trends Endocrinol. Metab..

[143] Evans C.O., Reddy P., Brat D.J., O’Neill E.B., Craige B., Stevens V.L., Oyesiku N.M. (2003). Differential expression of folate receptor in pituitary adenomas. Cancer Res..

[144] Ding Z., Li B., Wang Q., Miao Y., Lu X. (2015). Increase in folate receptor alpha expression in nonfunctional pituitary adenomas. Turk. Neurosurg..

[145] Liu X., Ma S., Yao Y., Li G., Feng M., Deng K., Dai C., Cai F., Li Y., Zhang B. (2012). Differential expression of folate receptor alpha in pituitary adenomas and its relationship to tumor behavior. Neurosurgery.

[146] Dai C., Cai F., Hwang K.C., Zhou Y., Zhang Z., Liu X., Ma S., Yang Y., Yao Y., Feng M. (2013). Folate receptor-mediated boron-10 containing carbon nanoparticles as potential delivery vehicles for boron neutron capture therapy of nonfunctional pituitary adenomas. Sci. China Life Sci..

[147] Larysz D., Zebracka-Gala J., Rudnik A., Hasse-Lazar K., Kowalska M., Jarząb M., Król A., Szpak-Ulczok S., Bażowski P., Jarząb B. (2012). Expression of genes FOLR1, BAG1 and LAPTM4B in functioning and non-functioning pituitary adenomas. Folia Neuropathol..

[148] Moreno C.S., Evans C.O., Zhan X., Okor M., Desiderio D.M., Oyesiku N.M. (2005). Novel molecular signaling and classification of human clinically nonfunctional pituitary adenomas identified by gene expression profiling and proteomic analyses. Cancer Res..

[149] Yao C., Evans C.O., Stevens V.L., Owens T.R., Oyesiku N.M. (2009). Folate receptor alpha regulates cell proliferation in mouse gonadotroph alphaT3-1 cells. Exp. Cell Res..

[150] Galt J.R., Halkar R.K., Evans C.O., Osman N.A., LaBorde D., Fox T.H., Faraj B.A., Kumar K., Wang H., Oyesiku N.M. (2010). In vivo assay of folate receptors in nonfunctional pituitary adenomas with 99mTc-folate SPECT/CT. J. Nucl. Med..

[151] Liu X., Ma S., Dai C., Cai F., Yao Y., Yang Y., Feng M., Deng K., Li G., Ma W. (2013). Antiproliferative, antiinvasive, and proapoptotic activity of folate receptor α-targeted liposomal doxorubicin in nonfunctional pituitary adenoma cells. Endocrinology.

[152] Paterni I., Granchi C., Katzenellenbogen J.A., Minutolo F. (2014). Estrogen receptors alpha (ERα) and beta (ERβ): Subtype-selective ligands and clinical potential. Steroids.

[153] Yaşar P., Ayaz G., User S.D., Güpür G., Muyan M. (2016). Molecular mechanism of estrogen-estrogen receptor signaling. Reprod. Med. Biol..

[154] Wang Z., Zhang X., Shen P., Loggie B.W., Chang Y., Deuel T.F. (2006). A variant of estrogen receptor-{alpha}, hER-{alpha}36: Transduction of estrogen- and antiestrogen-dependent membrane-initiated mitogenic signaling. Proc. Natl. Acad. Sci. USA.

[155] Wang C., Hu Z.Q., Chu M., Wang Z., Zhang W.G., Wang L.Z., Li C.G., Wang J.S. (2012). Resveratrol inhibited GH3 cell growth and decreased prolactin level via estrogen receptors. Clin. Neurol. Neurosurg..

[156] Hannen R., Steffani M., Voellger B., Carl B., Wang J., Bartsch J.W., Nimsky C. (2019). Effects of anti-estrogens on cell invasion and survival in pituitary adenoma cells: A systematic study. J. Steroid Biochem. Mol. Biol..

[157] Kansra S., Yamagata S., Sneade L., Foster L., Ben-Jonathan N. (2005). Differential effects of estrogen receptor antagonists on pituitary lactotroph proliferation and prolactin release. Mol. Cell Endocrinol..

[158] Gao H., Xue Y., Cao L., Liu Q., Liu C., Shan X., Wang H., Gu Y., Zhang Y. (2017). ESR1 and its antagonist fulvestrant in pituitary adenomas. Mol. Cell. Endocrinol..

[159] Wierinckx A., Delgrange E., Bertolino P., François P., Chanson P., Jouanneau E., Lachuer J., Trouillas J., Raverot G. (2018). Sex-Related Differences in Lactotroph Tumor Aggressiveness Are Associated with a Specific Gene-Expression Signature and Genome Instability. Front. Endocrinol..

[160] Trouillas J., Delgrange E., Wierinckx A., Vasiljevic A., Jouanneau E., Burman P., Raverot G. (2019). Clinical, Pathological, and Molecular Factors of Aggressiveness in Lactotroph Tumors. Neuroendocrinology.

[161] Delgrange E., Vasiljevic A., Wierinckx A., François P., Jouanneau E., Raverot G., Trouillas J. (2015). Expression of estrogen receptor alpha is associated with prolactin pituitary tumor prognosis and supports the sex-related difference in tumor growth. Eur. J. Endocrinol..

[162] Bima C., Chiloiro S., Giampietro A., Gessi M., Mattogno P.P., Lauretti L., Anile C., Rindi G., Pontecorvi A., De Marinis L. (2021). Galectin-3 and Estrogen Receptor Alpha as Prognostic Markers in Prolactinoma: Preliminary Results from a Pilot Study. Front. Endocrinol..

[163] Burdman J.A., Pauni M., Sereno G.M.H., Bordón A.E. (2008). Estrogen receptors in human pituitary tumors. Horm. Metab. Res..

[164] Li C.Z., Gui S.B., Zong X.Y., Zhang Y.Z. (2015). The Expression of Estrogen Receptor Subtypes in Prolactinomas and Their Relationship to Tumor Biological Behavior. Biomed. Env. Sci..

[165] Mahboobifard F., Bidari-Zerehpoosh F., Davoudi Z., Panahi M., Dargahi L., Pourgholami M.H., Sharifi G., Izadi N., Jorjani M. (2020). Expression patterns of ERα66 and its novel variant isoform ERα36 in lactotroph pituitary adenomas and associations with clinicopathological characteristics. Pituitary.

[166] Nishioka H., Tamura K., Iida H., Kutsukake M., Endo A., Ikeda Y., Haraoka J. (2011). Co-expression of somatostatin receptor subtypes and estrogen receptor-α mRNAs by non-functioning pituitary adenomas in young patients. Mol. Cell. Endocrinol..

[167] Manoranjan B., Salehi F., Scheithauer B.W., Rotondo F., Kovacs K., Cusimano M.D. (2010). Estrogen receptors alpha and beta immunohistochemical expression: Clinicopathological correlations in pituitary adenomas. Anticancer. Res..

[168] Su Y.X., Du G.L., Shen H.L., Wang W., Bao J.L., Aierken A., Wang B.W., Jiang S., Zhu J., Gao X.M. (2019). Increased expression of aromatase cytochrome P450 enzyme is associated with prolactinoma invasiveness in post-menopausal women. J. Int. Med. Res..

[169] Xiao Z., Yang X., Zhang K., Liu Z., Shao Z., Song C., Wang X., Li Z. (2020). Estrogen receptor α/prolactin receptor bilateral crosstalk promotes bromocriptine resistance in prolactinomas. Int. J. Med. Sci..

[170] Lv H., Li C., Gui S., Zhang Y. (2012). Expression of estrogen receptor α and growth factors in human prolactinoma and its correlation with clinical features and gender. J. Endocrinol. Investig..

[171] Zhou W., Song Y., Xu H., Zhou K., Zhang W., Chen J., Qin M., Yi H., Gustafsson J.A., Yang H. (2011). In nonfunctional pituitary adenomas, estrogen receptors and slug contribute to the development of invasiveness. J. Clin. Endocrinol. Metab..

[172] Pereira-Lima J.F., Marroni C.P., Pizarro C.B., Barbosa-Coutinho L.M., Ferreira N.P., Oliveira M.C. (2004). Immunohistochemical detection of estrogen receptor alpha in pituitary adenomas and its correlation with cellular replication. Neuroendocrinology.

[173] Øystese K.A., Casar-Borota O., Normann K.R., Zucknick M., Berg J.P., Bollerslev J. (2017). Estrogen Receptor α, a Sex-Dependent Predictor of Aggressiveness in Nonfunctioning Pituitary Adenomas: SSTR and Sex Hormone Receptor Distribution in NFPA. J. Clin. Endocrinol. Metab..

[174] Voellger B., Waldt N., Rupa R., Kirches E., Melhem O., Ochel H.J., Mawrin C., Firsching R. (2018). Combined effects of resveratrol and radiation in GH3 and TtT/GF pituitary adenoma cells. J. Neuro Oncol..

[175] Zhang Z., Bartsch J.W., Benzel J., Lei T., Nimsky C., Voellger B. (2020). Selective estrogen receptor modulators decrease invasiveness in pituitary adenoma cell lines AtT-20 and TtT/GF by affecting expression of MMP-14 and ADAM12. FEBS Open Bio..

[176] Voellger B., Kirches E., Wilisch-Neumann A., Weise A., Tapia-Perez J.H., Rupa R., Mawrin C., Firsching R. (2013). Resveratrol decreases B-cell lymphoma-2 expression and viability in GH3 pituitary adenoma cells of the rat. Onco Targets Ther..

[177] Wright D.J., Earnhardt J.N., Perry R., Bailey S., Komm B., Minck D.R., Cukierski M.A. (2013). Carcinogenicity and hormone studies with the tissue-selective estrogen receptor modulator bazadoxifene. J. Cell Physiol..

[178] Wang C., Bai M., Wang X., Tan C., Zhang D., Chang L., Li G., Xie L., Su J., Wen Y. (2018). Estrogen receptor antagonist fulvestrant inhibits proliferation and promotes apoptosis of prolactinoma cells by regulating the IRE1/XBP1 signaling pathway. Mol. Med. Rep..

[179] Dimaraki E.V., Symons K.V., Barkan A.L. (2004). Raloxifene decreases serum IGF-I in male patients with active acromegaly. Eur. J. Endocrinol..

[180] Choudhary C., Hamrahian A.H., Bena J.F., Recinos P., Kennedy L., Dobri G. (2019). The effect of raloxifene on serum prolactin level in patients with prolactinoma. Endocr. Pract..

[181] Ceccato F., Lizzul L., Voltan G., Barbot M., Scaroni C. (2021). Anastrozole as add-on therapy for cabergoline-resistant prolactin-secreting pituitary adenomas: Real-life experience in male patients. Pituitary.

[182] Balili I., Barkan A. (2014). Tamoxifen as a therapeutic agent in acromegaly. Pituitary.

[183] Liu J., Xiao Q., Xiao J., Niu C., Li Y., Zhang X., Zhou Z., Shu G., Yin G. (2022). Wnt/β-catenin signalling: Function, biological mechanisms, and therapeutic opportunities. Signal Transduct. Target. Ther..

[184] Tian X., Liu Z., Niu B., Zhang J., Tan T.K., Lee S.R., Zhao Y., Harris D.C., Zheng G. (2011). E-cadherin/β-catenin complex and the epithelial barrier. J. Biomed. Biotechnol..

[185] Chauvet N., Romanò N., Meunier A.C., Galibert E., Fontanaud P., Mathieu M.N., Osterstock G., Osterstock P., Baccino E., Rigau V. (2016). Combining Cadherin Expression with Molecular Markers Discriminates Invasiveness in Growth Hormone and Prolactin Pituitary Adenomas. J. Neuroendocr..

[186] Mendes G.A., Haag T., Trott G., Rech C.G.S.L., Ferreira N.P., Oliveira M.C., Kohek M.B., Pereira-Lima J.F.S. (2017). Expression of E-cadherin, Slug and NCAM and its relationship to tumor invasiveness in patients with acromegaly. Braz. J. Med. Biol. Res..

[187] Qian Z.R., Sano T., Yoshimoto K., Asa S.L., Yamada S., Mizusawa N., Kudo E. (2007). Tumor-specific downregulation and methylation of the CDH13 (H-cadherin) and CDH1 (E-cadherin) genes correlate with aggressiveness of human pituitary adenomas. Mod. Pathol..

[188] Øystese K.A.B., Casar-Borota O., Berg-Johnsen J., Berg J.P., Bollerslev J. (2022). Distribution of E- and N-cadherin in subgroups of non-functioning pituitary neuroendocrine tumors. Endocrine.

[189] Elston M.S., Gill A.J., Conaglen J.V., Clarkson A., Cook R.J., Little N.S., Robinson B.G., Clifton-Bligh R.J., McDonald K.L. (2009). Nuclear accumulation of e-cadherin correlates with loss of cytoplasmic membrane staining and invasion in pituitary adenomas. J. Clin. Endocrinol. Metab..

[190] Øystese K.A.B., Berg J.P., Normann K.R., Zucknick M., Casar-Borota O., Bollerslev J. (2018). The role of E and N-cadherin in the postoperative course of gonadotroph pituitary tumors. Endocrine.

[191] Zhou K., Jin H., Luo Y. (2013). Expression and significance of E-cadherin and β-catenins in pituitary adenoma. Int. J. Surg. Pathol..

[192] Liu C., Wu Y., Yu S., Bai J., Li C., Wu D., Zhang Y. (2017). Increased β catenin and c-myc expression predict aggressive growth of non-functioning pituitary adenomas: An assessment using a tissue microarray-based approach. Mol. Med. Rep..

[193] Wu Y., Bai J., Hong L., Liu C., Yu S., Yu G., Zhang Y. (2016). Low expression of secreted frizzled-related protein 2 and nuclear accumulation of β-catenin in aggressive nonfunctioning pituitary adenoma. Oncol. Lett..

[194] Demarchi G., Valla S., Perrone S., Chimento A., Bonadeo N., Vitale D.L., Spinelli F.M., Cervio A., Sevlever G., Alaniz L. (2022). β-Catenin is reduced in membranes of human prolactinoma cells and it is inhibited by temozolomide in prolactin secreting tumor models. Tumor Biol..

[195] Righi A., Jin L., Zhang S., Stilling G., Scheithauer B.W., Kovacs K., Lloyd R.V. (2010). Identification and consequences of galectin-3 expression in pituitary tumors. Mol. Cell Endocrinol..

[196] Riss D., Jin L., Qian X., Bayliss J., Scheithauer B.W., Young W.F., Vidal S., Kovacs K., Raz A., Lloyd R.V. (2003). Differential expression of galectin-3 in pituitary tumors. Cancer Res..

[197] Yoshii T., Fukumori T., Honjo Y., Inohara H., Kim H.R., Raz A. (2002). Galectin-3 phosphorylation is required for its anti-apoptotic function and cell cycle arrest. J. Biol. Chem..

[198] Righi A., Morandi L., Leonardi E., Farnedi A., Marucci G., Sisto A., Frank G., Faustini-Fustini M., Zoli M., Mazzatenta D. (2013). Galectin-3 expression in pituitary adenomas as a marker of aggressive behavior. Hum. Pathol..

[199] Dai D., Li Y., Lu Q., Yu L., Min W., Wang L., Cao Y., Yue Z. (2014). GAL3 protein expression is related to clinical features of prolactin-secreting pituitary microadenoma and predicts its recurrence after surgical treatment. Cell. Physiol. Biochem..

[200] Kawamoto H., Kawamoto K., Mizoue T., Uozumi T., Arita K., Kurisu K. (1996). Matrix metalloproteinase-9 secretion by human pituitary adenomas detected by cell immunoblot analysis. Acta Neurochir..

[201] Liu W., Kunishio K., Matsumoto Y., Okada M., Nagao S. (2005). Matrix metalloproteinase-2 expression correlates with cavernous sinus invasion in pituitary adenomas. J. Clin. Neurosci..

[202] Turner H.E., Nagy Z., Esiri M.M., Harris A.L., Wass J.A. (2000). Role of matrix metalloproteinase 9 in pituitary tumor behavior. J. Clin. Endocrinol. Metab..

[203] Gültekin G.D., Çabuk B., Vural Ç., Ceylan S. (2015). Matrix metalloproteinase-9 and tissue inhibitor of matrix metalloproteinase-2: Prognostic biological markers in invasive prolactinomas. J. Clin. Neurosci..

[204] Liu H., Zhang S., Wu T., Lv Z., Ba J., Gu W., Mu Y. (2022). Expression and clinical significance of Cathepsin K and MMPs in invasive non-functioning pituitary adenomas. Front. Oncol..

[205] Liu X., Feng M., Zhang Y., Dai C., Sun B., Bao X., Deng K., Yao Y., Wang R. (2018). Expression of Matrix Metalloproteinase-9, Pituitary Tumor Transforming Gene, High Mobility Group A 2, and Ki-67 in Adrenocorticotropic Hormone-Secreting Pituitary Tumors and Their Association with Tumor Recurrence. World Neurosurg..

[206] Gong J., Zhao Y., Abdel-Fattah R., Amos S., Xiao A., Lopes M.B., Hussaini I.M., Laws E.R. (2008). Matrix metalloproteinase-9, a potential biological marker in invasive pituitary adenomas. Pituitary.

[207] Hussaini I.M., Trotter C., Zhao Y., Abdel-Fattah R., Amos S., Xiao A., Agi C.U., Redpath G.T., Fang Z., Leung G.K. (2007). Matrix metalloproteinase-9 is differentially expressed in nonfunctioning invasive and noninvasive pituitary adenomas and increases invasion in human pituitary adenoma cell line. Am. J. Pathol..

[208] Beaulieu E., Kachra Z., Mousseau N., Delbecchi L., Hardy J., Béliveau R. (1999). Matrix metalloproteinases and their inhibitors in human pituitary tumors. Neurosurgery.

[209] Alvaro V., Lévy L., Dubray C., Roche A., Peillon F., Quérat B., Joubert D. (1993). Invasive human pituitary tumors express a point-mutated alpha-protein kinase-C. J. Clin. Endocrinol. Metab..

[210] Alvaro V., Touraine P., Vozari R.R., Bai-Grenier F., Birman P., Joubert D. (1992). Protein kinase C activity and expression in normal and adenomatous human pituitaries. Int. J. Cancer.

[211] Feng J., Yu S.Y., Li C.Z., Li Z.Y., Zhang Y.Z. (2016). Integrative proteomics and transcriptomics revealed that activation of the IL-6R/JAK2/STAT3/MMP9 signaling pathway is correlated with invasion of pituitary null cell adenomas. Mol. Cell. Endocrinol..

[212] Knappe U.J., Hagel C., Lisboa B.W., Wilczak W., Lüdecke D.K., Saeger W. (2003). Expression of serine proteases and metalloproteinases in human pituitary adenomas and anterior pituitary lobe tissue. Acta Neuropathol..

[213] Mao J.H., Guo H., Si N., Qiu L., Guo L.F., Sun Z.S., Xiang Y., Yang X.H., Zhao W.G., Zhang W.C. (2015). Regulating effect of MMP-9 and TIMP-1 in pituitary adenoma invasion. Genet. Mol. Res..

[214] Hui P., Xu X., Xu L., Hui G., Wu S., Lan Q. (2015). Expression of MMP14 in invasive pituitary adenomas: Relationship to invasion and angiogenesis. Int. J. Clin. Exp. Pathol..

[215] Deryugina E.I., Soroceanu L., Strongin A.Y. (2002). Up-regulation of vascular endothelial growth factor by membrane-type 1 matrix metalloproteinase stimulates human glioma xenograft growth and angiogenesis. Cancer Res..

[216] Yao G., He P., Chen L., Hu X., Gu F., Ye C. (2013). MT1-MMP in breast cancer: Induction of VEGF-C correlates with metastasis and poor prognosis. Cancer Cell Int..

[217] Ruskyte K., Liutkevicienė R., Vilkeviciute A., Vaitkiene P., Valiulytė I., Glebauskiene B., Kriauciuniene L., Zaliuniene D. (2016). MMP-14 and TGFβ-1 methylation in pituitary adenomas. Oncol. Lett..

[218] Winer A., Adams S., Mignatti P. (2018). Matrix Metalloproteinase Inhibitors in Cancer Therapy: Turning Past Failures into Future Successes. Mol. Cancer Ther..

[219] Matano F., Yoshida D., Ishii Y., Tahara S., Teramoto A., Morita A. (2014). Endocan, a new invasion and angiogenesis marker of pituitary adenomas. J. Neuro Oncol..

[220] Nomura R., Yoshida D., Teramoto A. (2009). Stromal cell-derived factor-1 expression in pituitary adenoma tissues and upregulation in hypoxia. J. Neurooncol..

[221] Niveiro M., Aranda F.I., Peiró G., Alenda C., Picó A. (2005). Immunohistochemical analysis of tumor angiogenic factors in human pituitary adenomas. Hum. Pathol..

[222] Sánchez-Ortiga R., Sánchez-Tejada L., Moreno-Perez O., Riesgo P., Niveiro M., Alfonso A.M.P. (2013). Over-expression of vascular endothelial growth factor in pituitary adenomas is associated with extrasellar growth and recurrence. Pituitary.

[223] Di Ieva A., Weckman A., Di Michele J., Rotondo F., Grizzi F., Kovacs K., Cusimano M.D. (2013). Microvascular morphometrics of the hypophysis and pituitary tumors: From bench to operating theatre. Microvasc. Res..

[224] Takada K., Yamada S., Teramoto A. (2004). Correlation between tumor vascularity and clinical findings in patients with pituitary adenomas. Endocr. Pathol..

[225] Vidal S., Kovacs K., Horvath E., Scheithauer B.W., Kuroki T., Lloyd R.V. (2001). Microvessel density in pituitary adenomas and carcinomas. Virchows Arch..

[226] Viacava P., Gasperi M., Acerbi G., Manetti L., Cecconi E., Bonadio A.G., Naccarato A.G., Acerbi F., Parenti G., Lupi I. (2003). Microvascular density and vascular endothelial growth factor expression in normal pituitary tissue and pituitary adenomas. J. Endocrinol. Investig..

[227] Roskoski R. (2008). VEGF receptor protein-tyrosine kinases: Structure and regulation. Biochem. Biophys. Res. Commun..

[228] Nör J.E., Christensen J., Mooney D.J., Polverini P.J. (1999). Vascular endothelial growth factor (VEGF)-mediated angiogenesis is associated with enhanced endothelial cell survival and induction of Bcl-2 expression. Am. J. Pathol..

[229] Dai C., Liang S., Sun B., Li Y., Kang J. (2021). Anti-VEGF Therapy in Refractory Pituitary Adenomas and Pituitary Carcinomas: A Review. Front. Oncol..

[230] Yilmaz M., Vural E., Koc K., Ceylan S. (2015). Cavernous sinus invasion and effect of immunohistochemical features on remission in growth hormone secreting pituitary adenomas. Turk. Neurosurg..

[231] Borg S.A., Kerry K.E., Royds J.A., Battersby R.D., Jones T.H. (2005). Correlation of VEGF production with IL1 alpha and IL6 secretion by human pituitary adenoma cells. Eur. J. Endocrinol..

[232] Ghadir M., Khamseh M.E., Panahi-Shamsabad M., Ghorbani M., Akbari H., Mehrjardi A.Z., Honardoost M., Jafar-Mohammadi B. (2020). Cell proliferation, apoptosis, and angiogenesis in non-functional pituitary adenoma: Association with tumor invasiveness. Endocrine.

[233] Wang Y., Li J., Tohti M., Hu Y., Wang S., Li W., Lu Z., Ma C. (2014). The expression profile of Dopamine D2 receptor, MGMT and VEGF in different histological subtypes of pituitary adenomas: A study of 197 cases and indications for the medical therapy. J. Exp. Clin. Cancer Res..

[234] Cristina C., Díaz-Torga G., Baldi A., Góngora A., Rubinstein M., Low M.J., Becú-Villalobos D. (2005). Increased pituitary vascular endothelial growth factor-a in dopaminergic D2 receptor knockout female mice. Endocrinology.

[235] Korsisaari N., Ross J., Wu X., Kowanetz M., Pal N., Hall L., Eastham-Anderson J., Forrest W.F., Van Bruggen N., Peale F.V. (2008). Blocking vascular endothelial growth factor-A inhibits the growth of pituitary adenomas and lowers serum prolactin level in a mouse model of multiple endocrine neoplasia type 1. Clin. Cancer Res..

[236] Zhou J., Hu Y., Zhu W., Nie C., Zhao W., Faje A.T., Labelle K.E., Swearingen B., Lee H., Hedley-Whyte E.T. (2022). Sprouting Angiogenesis in Human Pituitary Adenomas. Front. Oncol..

[237] Miyajima K., Takekoshi S., Itoh J., Kakimoto K., Miyakoshi T., Osamura R.Y. (2010). Inhibitory effects of anti-VEGF antibody on the growth and angiogenesis of estrogen-induced pituitary prolactinoma in Fischer 344 Rats: Animal model of VEGF-targeted therapy for human endocrine tumors. Acta Histochem. Cytochem..

[238] Kurosaki M., Saegert W., Abe T., Lüdecke D.K. (2008). Expression of vascular endothelial growth factor in growth hormone-secreting pituitary adenomas: Special reference to the octreotide treatment. Neurol. Res..

[239] Amato R., Catalani E., Monte M.D., Cammalleri M., Di Renzo I., Perrotta C., Cervia D., Casini G. (2018). Autophagy-mediated neuroprotection induced by octreotide in an ex vivo model of early diabetic retinopathy. Pharmacol. Res..

[240] Pan K.F., Yang Y.C., Lee W.J., Hua K.T., Chien M.H. (2022). Proteoglycan Endocan: A multifaceted therapeutic target in Cancer. Biochim. Biophys. Acta Rev. Cancer.

[241] Miao Y., Zong M., Jiang T., Yuan X., Guan S., Wang Y., Zhou D. (2016). A comparative analysis of ESM-1 and vascular endothelial cell marker (CD34/CD105) expression on pituitary adenoma invasion. Pituitary.

[242] Cornelius A., Cortet-Rudelli C., Assaker R., Kerdraon O., Gevaert M.H., Prévot V., Lassalle P., Trouillas J., Delehedde M., Maurage C.A. (2012). Endothelial expression of endocan is strongly associated with tumor progression in pituitary adenoma. Brain Pathol..

[243] Wang S., Wu Z., Wei L., Zhang J. (2019). Endothelial cell-specific molecule-1 as an invasiveness marker for pituitary null cell adenoma. BMC Endocr. Disord..

[244] Tao P., Liu X., Zhang Q., Chen G., Ling F. (2022). Associations of Endocan, FGF2, and PDGF Expression with Pituitary Neuroendocrine Tumor (PitNET) Invasiveness. Turk. Neurosurg..

[245] Roudnicky F., Poyet C., Wild P., Krampitz S., Negrini F., Huggenberger R., Rogler A., Stöhr R., Hartmann A., Provenzano M. (2013). Endocan is upregulated on tumor vessels in invasive bladder cancer where it mediates VEGF-A-induced angiogenesis. Cancer Res..

[246] Tsai H.F., Chang Y.C., Li C.H., Chan M.H., Chen C.L., Tsai W.C., Hsiao M. (2021). Type V collagen alpha 1 chain promotes the malignancy of glioblastoma through PPRC1-ESM1 axis activation and extracellular matrix remodeling. Cell Death Discov..

[247] Sun L., Sun C., Sun J., Yang W. (2019). Downregulation of ENDOCAN in myeloid leukemia cells inhibits proliferation and promotes apoptosis by suppressing nuclear factor κB activity. Mol. Med. Rep..

[248] Cai L., Leng Z.G., Guo Y.H., Lin S.J., Wu Z.R., Su Z.P., Lu J.L., Wei L.F., Zhuge Q.C., Jin K. (2016). Dopamine agonist resistance-related endocan promotes angiogenesis and cells viability of prolactinomas. Endocrine.

[249] Vierimaa O., Georgitsi M., Lehtonen R., Vahteristo P., Kokko A., Raitila A., Tuppurainen K., Ebeling T.M., Salmela P.I., Paschke R. (2006). Pituitary adenoma predisposition caused by germline mutations in the AIP gene. Science.

[250] Re A., Ferraù F., Cafiero C., Spagnolo F., Barresi V., Romeo D.P., Ragonese M., Grassi C., Pontecorvi A., Farsetti A. (2020). Somatic Deletion in Exon 10 of Aryl Hydrocarbon Receptor Gene in Human GH-Secreting Pituitary Tumors. Front. Endocrinol..

[251] De Pinho L.K.J., Neto L.V., Wildemberg L.E.A., Gasparetto E.L., Marcondes J., de Almeida Nunes B., Takiya C.M., Gadelha M.R. (2011). Low aryl hydrocarbon receptor-interacting protein expression is a better marker of invasiveness in somatotropinomas than Ki-67 and p53. Neuroendocrinology.

[252] Bogner E.M., Daly A.F., Gulde S., Karhu A., Irmler M., Beckers J., Mohr H., Beckers A., Pellegata N.S. (2020). miR-34a is upregulated in AIP-mutated somatotropinomas and promotes octreotide resistance. Int. J. Cancer.

[253] Ritvonen E., Pitkänen E., Karppinen A., Vehkavaara S., Demir H., Paetau A., Schalin-Jäntti C., Karhu A. (2017). Impact of AIP and inhibitory G protein alpha 2 proteins on clinical features of sporadic GH-secreting pituitary adenomas. Eur. J. Endocrinol..

[254] Dutta P., Reddy K.S., Rai A., Madugundu A.K., Solanki H.S., Bhansali A., Radotra B.D., Kumar N., Collier D., Iacovazzo D. (2019). Surgery, Octreotide, Temozolomide, Bevacizumab, Radiotherapy, and Pegvisomant Treatment of an AIP Mutation–Positive Child. J. Clin. Endocrinol. Metab..

[255] Cai F., Chen S., Yu X., Zhang J., Liang W., Zhang Y., Chen Y., Chen S., Hong Y., Yan W. (2022). Transcription factor GTF2B regulates AIP protein expression in growth hormone-secreting pituitary adenomas and influences tumor phenotypes. Neuro Oncol..

[256] Neou M., Villa C., Armignacco R., Jouinot A., Raffin-Sanson M.L., Septier A., Letourneur F., Diry S., Diedisheim M., Izac B. (2020). Pangenomic Classification of Pituitary Neuroendocrine Tumors. Cancer Cell.

[257] Sapkota S., Horiguchi K., Tosaka M., Yamada S., Yamada M. (2017). Whole-Exome Sequencing Study of Thyrotropin-Secreting Pituitary Adenomas. J. Clin. Endocrinol. Metab..

[258] Zhang F., Zhang Q., Zhu J., Yao B., Ma C., Qiao N., He S., Ye Z., Wang Y., Han R. (2022). Integrated proteogenomic characterization across major histological types of pituitary neuroendocrine tumors. Cell Res..

[259] Landis C.A., Harsh G., Lyons J., Davis R.L., McCormick F., Bourne H.R. (1990). Clinical characteristics of acromegalic patients whose pituitary tumors contain mutant Gs protein. J. Clin. Endocrinol. Metab..

[260] Buchfelder M., Fahlbusch R., Merz T., Symowski H., Adams E.F. (2007). Analysis of GNAS mutations in 60 growth hormone secreting pituitary tumors: Correlation with clinical and pathological characteristics and surgical outcome based on highly sensitive GH and IGF-I criteria for remission. Pituitary.

[261] Buchfelder M., Fahlbusch R., Merz T., Symowski H., Adams E.F. (1999). Clinical correlates in acromegalic patients with pituitary tumors expressing GSP oncogenes. Pituitary.

[262] Efstathiadou Z.A., Bargiota A., Chrisoulidou A., Kanakis G., Papanastasiou L., Theodoropoulou A., Tigas S.K., Vassiliadi D.A., Alevizaki M., Tsagarakis S. (2015). Impact of gsp mutations in somatotroph pituitary adenomas on growth hormone response to somatostatin analogs: A meta-analysis. Pituitary.

[263] Foltran R.K., Amorim P.V.G.H., Duarte F.H., Grande I.P.P., Freire A.C.T.B., Frassetto F.P., Dettoni J.B., Alves V.A., Castro I., Trarbach E.B. (2018). Study of major genetic factors involved in pituitary tumorigenesis and their impact on clinical and biological characteristics of sporadic somatotropinomas and non-functioning pituitary adenomas. Braz. J. Med. Biol. Res..

[264] Niendorf S., Oksche A., Kisser A., Löhler J., Prinz M., Schorle H., Feller S., Lewitzky M., Horak I., Knobeloch K.P. (2007). Essential role of ubiquitin-specific protease 8 for receptor tyrosine kinase stability and endocytic trafficking in vivo. Mol. Cell. Biol..

[265] Byun S., Lee S.Y., Lee J., Jeong C.H., Farrand L., Lim S., Reddy K., Kim J.Y., Lee M.H., Lee H.J. (2013). USP8 is a novel target for overcoming gefitinib resistance in lung cancer. Clin. Cancer Res..

[266] Ma Z.Y., Song Z.J., Chen J.H., Wang Y.F., Li S.Q., Zhou L.F., Mao Y., Li Y.M., Hu R.G., Zhang Z.Y. (2015). Recurrent gain-of-function USP8 mutations in Cushing’s disease. Cell Res..

[267] Perez-Rivas L.G., Theodoropoulou M., Ferraù F., Nusser C., Kawaguchi K., Stratakis C.A., Faucz F.R., Wildemberg L.E., Assié G., Beschorner R. (2015). The Gene of the Ubiquitin-Specific Protease 8 Is Frequently Mutated in Adenomas Causing Cushing’s Disease. J. Clin. Endocrinol. Metab..

[268] Albani A., Pérez-Rivas L.G., Dimopoulou C., Zopp S., Colón-Bolea P., Roeber S., Honegger J., Flitsch J., Rachinger W., Buchfelder M. (2018). The USP8 mutational status may predict long-term remission in patients with Cushing’s disease. Clin. Endocrinol..

[269] Faucz F.R., Tirosh A., Tatsi C., Berthon A., Hernández-Ramírez L.C., Settas N., Angelousi A., Correa R., Papadakis G.Z., Chittiboina P. (2017). Somatic USP8 Gene Mutations Are a Common Cause of Pediatric Cushing Disease. J. Clin. Endocrinol. Metab..

[270] Wanichi I.Q., de Paula Mariani B.M., Frassetto F.P., Siqueira S.A.C., de Castro Musolino N.R., Cunha-Neto M.B.C., Ochman G., Cescato V.A.S., Machado M.C., Trarbach E.B. (2019). Cushing’s disease due to somatic USP8 mutations: A systematic review and meta-analysis. Pituitary.

[271] Hayashi K., Inoshita N., Kawaguchi K., Ardisasmita A.I., Suzuki H., Fukuhara N., Okada M., Nishioka H., Takeuchi Y., Komada M. (2016). The USP8 mutational status may predict drug susceptibility in corticotroph adenomas of Cushing’s disease. Eur. J. Endocrinol..

[272] Bruns C., Lewis I., Briner U., Meno-Tetang G., Weckbecker G. (2002). SOM230: A novel somatostatin peptidomimetic with broad somatotropin release inhibiting factor (SRIF) receptor binding and a unique antisecretory profile. Eur. J. Endocrinol..

[273] Duong C.V., Emes R.D., Wessely F., Yacqub-Usman K., Clayton R.N., Farrell W.E. (2012). Quantitative, genome-wide analysis of the DNA methylome in sporadic pituitary adenomas. Endocr. Relat. Cancer.

[274] Kober P., Boresowicz J., Rusetska N., Maksymowicz M., Goryca K., Kunicki J., Bonicki W., Siedlecki J.A., Bujko M. (2018). DNA methylation profiling in nonfunctioning pituitary adenomas. Mol. Cell Endocrinol..

[275] Dudley K.J., Revill K., Clayton R.N., Farrell W.E. (2009). Pituitary tumors: All silent on the epigenetics front. J. Mol. Endocrinol..

[276] Ling C., Pease M., Shi L., Punj V., Shiroishi M.S., Commins D., Weisenberger D.J., Wang K., Zada G. (2014). A pilot genome-scale profiling of DNA methylation in sporadic pituitary macroadenomas: Association with tumor invasion and histopathological subtype. PLoS ONE.

[277] Cheng S., Li C., Xie W., Miao Y., Guo J., Wang J., Zhang Y. (2020). Integrated analysis of DNA methylation and mRNA expression profiles to identify key genes involved in the regrowth of clinically non-functioning pituitary adenoma. Aging.

[278] Wang R.Q., Lan Y.L., Lou J.C., Lyu Y.Z., Hao Y.C., Su Q.F., Ma B.B., Yuan Z.B., Yu Z.K., Zhang H.Q. (2019). Expression and methylation status of LAMA2 are associated with the invasiveness of nonfunctioning PitNET. Ther. Adv. Endocrinol. Metab..

[279] Gadelha M.R., Kasuki L., Dénes J., Trivellin G., Korbonits M. (2013). MicroRNAs: Suggested role in pituitary adenoma pathogenesis. J. Endocrinol. Investig..

[280] Nadhamuni V.S., Korbonits M. (2020). Novel Insights into Pituitary Tumorigenesis: Genetic and Epigenetic Mechanisms. Endocr. Rev..

[281] Zhang T., Yang Z., Gao H. (2017). Advancements in the study of miRNA regulation during the cell cycle in human pituitary adenomas. J. Neurooncol.

[282] Renjie W., Haiqian L. (2015). MiR-132, miR-15a and miR-16 synergistically inhibit pituitary tumor cell proliferation, invasion and migration by targeting Sox5. Cancer Lett..

[283] Du Q., Zhang W., Feng Q., Hao B., Cheng C., Cheng Y., Li Y., Fan X., Chen Z. (2020). Comprehensive circular RNA profiling reveals that hsa_circ_0001368 is involved in growth hormone-secreting pituitary adenoma development. Brain Res. Bull..

[284] Shen D.W., Li Y.L., Hou Y.J., Xu Z.D., Li Y.Z., Chang J.Y. (2019). MicroRNA-543 promotes cell invasion and impedes apoptosis in pituitary adenoma via activating the Wnt/β-catenin pathway by negative regulation of Smad7. Biosci. Biotechnol. Biochem..

[285] Roche M., Wierinckx A., Croze S., Rey C., Legras-Lachuer C., Morel A.P., Fusco A., Raverot G., Trouillas J., Lachuer J. (2015). Deregulation of miR-183 and KIAA0101 in Aggressive and Malignant Pituitary Tumors. Front. Med..

[286] Garbicz F., Mehlich D., Rak B., Sajjad E., Maksymowicz M., Paskal W., Zieliński G., Włodarski P.K. (2017). Increased expression of the microRNA 106b~25 cluster and its host gene MCM7 in corticotroph pituitary adenomas is associated with tumor invasion and Crooke’s cell morphology. Pituitary.

[287] Zheng Z., Zhang Y., Zhang Z., Yang Y., Song T. (2017). Effect of miR-106b on Invasiveness of Pituitary Adenoma via PTEN-PI3K/AKT. Med. Sci. Monit..

[288] Palumbo T., Faucz F.R., Azevedo M., Xekouki P., Iliopoulos D., Stratakis C.A. (2013). Functional screen analysis reveals miR-26b and miR-128 as central regulators of pituitary somatomammotrophic tumor growth through activation of the PTEN-AKT pathway. Oncogene.

[289] Duan J., Lu G., Li Y., Zhou S., Zhou D., Tao H. (2017). Increased miR-338-3p expression correlates with invasiveness of GH-producing pituitary adenomas. Endocrine.

[290] Duan J., Lu G., Li Y., Zhou S., Zhou D., Tao H. (2019). miR-137 functions as a tumor suppressor gene in pituitary adenoma by targeting AKT2. Int. J. Clin. Exp. Pathol..

[291] Lei C., Jing G., Jichao W., Xiaohui L., Fang Q., Hua G., Yazhou M., Zhang Y. (2019). MiR-137’s Tumor Suppression on Prolactinomas by Targeting MITF and Modulating Wnt Signaling Pathway. J. Clin. Endocrinol. Metab..

[292] Song Z.J., Reitman Z.J., Ma Z.Y., Chen J.H., Zhang Q.L., Shou X.F., Huang C.X., Wang Y.F., Li S.Q., Mao Y. (2016). The genome-wide mutational landscape of pituitary adenomas. Cell Res..

[293] Grzywa T.M., Klicka K., Rak B., Mehlich D., Garbicz F., Zieliński G., Maksymowicz M., Sajjad E., Włodarski P.K. (2019). Lineage-dependent role of miR-410-3p as oncomiR in gonadotroph and corticotroph pituitary adenomas or tumor suppressor miR in somatotroph adenomas via MAPK, PTEN/AKT, and STAT3 signaling pathways. Endocrine.

[294] Lloyd R.V., Osamura R.Y., Klöppel G., Rosai J. (2017). Chapter 01: Tumours of the pituitary gland. WHO Classification of Tumours of Endocrine Organs.

[295] Mete O., Osamura R.Y., Asa L.S. (2022). Chapter 02: Pituitary Tumours. WHO Classification of Tumours Editorial Board. Endocrine and Neuroendocrine Tumours.

[296] Villa C., Baussart B., Assié G., Raverot G., Roncaroli F. (2023). The World Health Organization classifications of pituitary neuroendocrine tumours: A clinico-pathological appraisal. Endocr. Relat. Cancer.

[297] Lenders N.F., Earls P.E., Inder W.J., McCormack A.I. (2023). The evolution in pituitary tumour classification: A clinical perspective. Endocr. Oncol..

[298] Daly A.F., Tichomirowa M.A., Petrossians P., Heliövaara E., Jaffrain-Rea M.L., Barlier A., Naves L.A., Ebeling T., Karhu A., Raappana A. (2010). Clinical characteristics and therapeutic responses in patients with germ-line AIP mutations and pituitary adenomas: An international collaborative study. J. Clin. Endocrinol. Metab..

[299] Chiloiro S., De Marinis L. (2021). From Pituitary Adenoma to Pituitary Neuroendocrine Tumors: How Molecular Pathways may Impact the Therapeutic Management?. Endocr. Metab. Immune Disord. Drug Targets.

